# Effects of grid spacing on high-frequency precipitation variance in coupled high-resolution global ocean–atmosphere models

**DOI:** 10.1007/s00382-022-06257-6

**Published:** 2022-03-29

**Authors:** Charles X. Light, Brian K. Arbic, Paige E. Martin, Laurent Brodeau, J. Thomas Farrar, Stephen M. Griffies, Ben P. Kirtman, Lucas C. Laurindo, Dimitris Menemenlis, Andrea Molod, Arin D. Nelson, Ebenezer Nyadjro, Amanda K. O’Rourke, Jay F. Shriver, Leo Siqueira, R. Justin Small, Ehud Strobach

**Affiliations:** 1 Electrical Engineering and Computer Science Department, University of Michigan, Ann Arbor, MI USA; 2 Department of Earth and Environmental Sciences, University of Michigan, Ann Arbor, MI USA; 3 Department of Physics, University of Michigan, Ann Arbor, MI USA; 4 Lamont-Doherty Earth Observatory, Columbia University, Palisades, NY USA; 5 Research School of Earth Sciences, Australian National University, Canberra, Australia; 6 Institut des Géosciences de L’Environnement, CNRS-UGA, Grenoble, France; 7 Department of Physical Oceanography, Woods Hole Oceanographic Institution, Woods Hole, MA USA; 8 Geophysical Fluid Dynamics Laboratory, National Oceanic and Atmospheric Administration, + Princeton University Atmospheric and Oceanic Science Program, Princeton, NJ USA; 9 Rosenstiel School of Marine and Atmospheric Science, University of Miami, Miami, FL USA; 10 National Center for Atmospheric Research, Boulder, CO USA; 11 Jet Propulsion Laboratory, California Institute of Technology, Pasadena, CA USA; 12 NASA Goddard Space Flight Center, Greenbelt, MD USA; 13 Graduate School of Oceanography, University of Rhode Island, Narragansett, RI USA; 14 Northern Gulf Institute, Mississippi State University, Stennis Space Center, Hancock County, MS USA; 15 Applied Physics Laboratory, Johns Hopkins University, Laurel, MD USA; 16 Oceanographic Division, Naval Research Laboratory, Stennis Space Center, Hancock County, MS USA; 17 CMNS-Earth System Science Interdisciplinary Center, University of Maryland, College Park, MD USA; 18 Agricultural Research Organization, Volcani Institute, Rishon LeTsiyon, Israel

**Keywords:** Precipitation, High-frequency precipitation, Numerical modeling, High-resolution models, Coupled ocean-atmosphere models

## Abstract

High-frequency precipitation variance is calculated in 12 different free-running (non-data-assimilative) coupled high resolution atmosphere–ocean model simulations, an assimilative coupled atmosphere–ocean weather forecast model, and an assimilative reanalysis. The results are compared with results from satellite estimates of precipitation and rain gauge observations. An analysis of irregular sub-daily fluctuations, which was applied by Covey et al. (Geophys Res Lett 45:12514–12522, 2018. 10.1029/2018GL078926) to satellite products and low-resolution climate models, is applied here to rain gauges and higher-resolution models. In contrast to lower-resolution climate simulations, which Covey et al. (2018) found to be lacking with respect to variance in irregular sub-daily fluctuations, the highest-resolution simulations examined here display an irregular sub-daily fluctuation variance that lies closer to that found in satellite products. Most of the simulations used here cannot be analyzed via the Covey et al. (2018) technique, because they do not output precipitation at sub-daily intervals. Thus the remainder of the paper focuses on frequency power spectral density of precipitation and on cumulative distribution functions over time scales (2–100 days) that are still relatively “high-frequency” in the context of climate modeling. Refined atmospheric or oceanic model grid spacing is generally found to increase high-frequency precipitation variance in simulations, approaching the values derived from observations. Mesoscale-eddy-rich ocean simulations significantly increase precipitation variance only when the atmosphere grid spacing is sufficiently fine (< 0.5°). Despite the improvements noted above, all of the simulations examined here suffer from the “drizzle effect”, in which precipitation is not temporally intermittent to the extent found in observations.

## Introduction

Precipitation is a key variable in climate and weather models, and the skill of models in predicting precipitation is continually being assessed (United States Department of Energy 2020). Assessment of precipitation in climate models has often been performed for seasonal and longer time scales. However, precipitation is highly intermittent in space and time, and large events occurring over subseasonal timescales (with periods less than approximately 100 days) can have an inordinately high influence on precipitation totals (e.g., Trenberth et al. 2017; Covey et al. 2018). As Trenberth et al. (2003) point out, while long-term averages of precipitation in models and observations are often documented, the intensity, frequency, and duration of precipitation at high frequencies is seldom analyzed. More recently, Trenberth et al. (2017) and Covey et al. (2018) examined high-frequency precipitation in newer observational products, but pointed out that precipitation intermittency is still poorly simulated in numerical models, despite the fact that an “overwhelming fraction of precipitation variance comes from day-to-day variations at each hour of the diurnal cycle”.

In this paper, we focus attention on high-frequency precipitation in coupled atmosphere–ocean models as well as the importance of spatial resolution (grid spacing) in the atmospheric and oceanic components for the modeling of high-frequency precipitation. We focus on periods that are less than 100 days, and much less when the frequency of outputs in available models and observational products allows. We examine the impacts of model grid spacing and output frequency, due to the importance of spatial and temporal scales in the study of precipitation. Many atmospheric processes (particularly convection) that govern the formation of precipitation events occur at spatial scales much smaller than synoptic or global scales (Small et al. 2014). Therefore, we expect the high-frequency precipitation behavior in atmospheric models to depend on model grid spacing. Model output frequencies are also important; large individual precipitation events can occur on timescales as short as hours (Covey et al. 2018), much shorter than the output time scales of many climate models. Until recently, data storage limitations have resulted in many climate model simulations having outputs limited to monthly means. With the advent of larger supercomputers and storage silos, it is becoming more feasible to store modeled precipitation at higher frequencies. Supercomputer power also permits atmospheric models to be run with finer computational grids that come closer to resolving the spatial scales involved in high-frequency precipitation events. Higher temporal resolution also allows models to simulate extreme weather events and allows assessment of the sensitivity of such extreme events to climate change (Small et al. 2014).

We also examine the impact of ocean model grid spacing on high-frequency precipitation. Coupled atmosphere–ocean models are a workhorse tool for climate science, but until recently the grid spacings in the ocean component of coupled climate models have been coarse. Over the last decade or so, there has been increasing interest in high-resolution coupled atmosphere–ocean models (e.g., McClean et al. 2011; Kirtman et al., 2012; Small et al. 2014; Griffies et al. 2015; Chang et al. 2020), inspiring a new frontier in climate science for the modeling of climate and weather. Ocean models with coarse grid spacing miss the most energetic flows in the ocean, namely mesoscale eddies on scales of 25–500 km (e.g., Maltrud and McClean 2005; Hecht and Hasumi 2008; McClean et al. 2011). The sea surface temperature signal of mesoscale eddies can impact the temperature of the air above them, and hence drive convection and precipitation in the atmosphere directly above the ocean (Frenger et al. 2013).

We analyze outputs from a variety of modeling centers. The nominal atmospheric grid spacing in the models examined here ranges from 1/16° to 1/2°. As a point of reference, Trenberth et al. (2017) focused on CESM simulations with ~ 1° horizontal grid spacing. The ocean component in most of the coupled atmosphere–ocean models analyzed here is “eddying” (allows for a vigorous mesoscale eddy field; Hecht and Hasumi 2008), with grid spacings ranging from 1/10° to 1/25°. In some cases, we are able to analyze simulations for which either the atmosphere or ocean component alone is varied, allowing for a particularly direct discussion of the impacts of model grid spacing. One of the high-resolution atmosphere–ocean models employs data assimilation in both fluids, allowing for an assessment of the value added by assimilating data into climate and weather models. The modeling results explored in this work will be compared to a reanalysis, two satellite-based observational products, and to local rain gauges.

We analyze high-frequency precipitation in high-resolution coupled atmosphere–ocean models using three primary tools. First, we analyze the satellite products, rain gauges, and models for which subdaily outputs are provided with a technique used by Covey et al. (2018), who demonstrated that high-frequency precipitation variance is dominated by what they call “irregular sub-daily fluctuations” (fluctuations at periods that are sub-daily and are not harmonics of 24 h). Furthermore, models run with coarse grid spacings typical of many climate models have insufficient variance in the irregular sub-daily fluctuations relative to observations. Because most of the models analyzed here do not output precipitation at sub-daily intervals, we focus the remainder of the paper on analyses using other tools, and on timescales (2–100 days) that are longer than daily but are still high-frequency by the standards of climate modeling.

We use frequency-domain power spectral density (hereinafter, often shortened to “spectra” or “power spectra”) to display the precipitation variance as a function of frequency. Agreement of modeled power spectra with observations at a particular frequency is a necessary but not sufficient condition for concluding that the model offers an accurate rendition of observations. For example, two power spectra can agree even if the temporal phases disagree (e.g., Armi and Flament 1985). Time-domain analysis of other atmospheric models has identified cases for which the phase is systematically biased but in which the magnitude appears correct; one example is convective activity over tropical regions, which has been simulated too early in the daily cycle in some models (Flato et al. 2013). There has been previous work with power spectral density of precipitation rate to evaluate its usefulness in forecasting (Luque-Espinar et al. 2017), although that work focused on much longer timescales (about 150 days and longer, out to the 11-year sunspot cycle).

We also employ cumulative distribution functions (CDFs) of precipitation in our study. The atmospheric science community has employed CDFs more often than frequency spectra in the analysis of precipitation (e.g., United States Department of Energy 2020). Showing CDFs in conjunction with variance spectra is valuable because we can view both the sizes of precipitation events that occur and the amount of total precipitation associated with particular frequencies. CDFs are particularly good at quantifying the “drizzle effect” in which models have low amplitude and frequent rain events, more so than is found in observations.

In this paper, we investigate the effect of atmosphere and ocean model grid spacing on precipitation with a few key questions in mind: (1) Does refining atmospheric model grid spacing, and refining ocean model grid spacing to a mesoscale eddying level, yield high-frequency irregular sub-daily fluctuations, precipitation spectra and CDFs that better match observational data? (2) Do we see the same trends in model grid spacing and precipitation behavior across multiple model groups and regions? We also address the idea that spectral analysis may help with the goal of longer-term precipitation forecasting. While some analysis involving power spectra of precipitation rate has been done in the past (Luque-Espinar et al. 2017), no large-scale comparison of this kind has yet been conducted with the current generation of high-resolution coupled atmosphere–ocean models.

## Datasets

We examine high-frequency precipitation rates across two global observational estimates derived primarily from satellite data, two sets of rain gauge data over eight locations, one global reanalysis, one global atmosphere–ocean coupled weather forecast model, and 12 different global free-running atmosphere–ocean simulations. After displaying time series from the different models and observational datasets at a particular location, we examine irregular sub-daily fluctuations (Covey et al. 2018) in rain gauges, satellite products, and simulations for which precipitation is put out on sub-daily timescales. We then focus on frequency spectra and cumulative distribution functions (CDFs), over timescales (2–100 days) that are longer than daily but still “high frequency” relative to the longer-term fluctuations typically examined in the climate modeling literature. The 2–100 day mid-to-high frequency seasonal to sub-seasonal patterns of precipitation highlight the impact of oceanic mesoscale eddies. In order to fully resolve oceanic mesoscale eddy effects, the precipitation data should have high frequency output (of order once per day, preferably more often), and should be of sufficient length (preferably ~ 1 year or more) to cover several oceanic mesoscale eddy decorrelation times, which are about 35 days.

Where available, 5 years of output data is analyzed to allow for accurate representation of power spectral density at seasonal and sub-seasonal scales. There are some exceptions to this 5-year output duration. We analyze four model simulations and two rain gauges for which we only have 1 year of output. In addition, for the higher-resolution GEOS/ECCO model (see below), we only have 84 days of output. In analyses performed on this 84-day dataset, we must pay attention to the lack of a complete annual cycle.

While the majority of products analyzed in this paper output precipitation accumulation, two products (the TRMM dataset and the GEOS/ECCO 3-month higher-resolution run) instead output snapshots of precipitation rate, a difference that manifests as a change in slope at the highest frequencies (representing time scales far shorter than the daily cycle) seen in frequency spectra.

### TRMM

We use the NASA Tropical Rainfall Measuring Mission (TRMM) 3B42 3-hourly satellite observational product. TRMM recorded estimates of precipitation rate for the years 1998–2014 between 50° N and 50° S latitude at a grid spacing of 0.25°. The TRMM product uses rain gauge data for calibration (Huffman et al. 2007). We place much of our focus on oceanic regions where there is a lower volume of gauges to compare against. Because the TRMM mission was focused on measuring precipitation in tropical and subtropical latitudes, the dataset does have some weaknesses (Trenberth et al. 2017) at temperate latitudes. This data is in the public domain and is available to download at http://disc.gsfc.nasa.gov/datasets/TRMM_3B42_V7/summary?keywords=TRMM_3B42 and http://mirador.gsfc.nasa.gov.

### CMORPH

We also use the Climate Prediction Center Morphing Technique (CMORPH) product developed by NOAA (Xie et al. 2017). The estimate is based on satellite observations, with calibration provided by ground-based precipitation measurements (e.g., Covey et al. 2018) and machine learning algorithms used to process the raw data into a usable product. While every observational product has advantages and disadvantages, CMORPH is often considered to produce more realistic precipitation fields than the TRMM product, particularly outside of tropical regions (Tapiador et al. 2017). Hence, we consider CMORPH as our benchmark global observational dataset in this work, and complement it with other observational datasets (TRMM and rain gauges). We use a CMORPH product with 0.25° grid spacing and 3-h output frequency such as to be consistent with the TRMM dataset. CMORPH originally started processing data from December 2002, although a reprocessed version (v1.0) has a start date of 1998 to align with the start of the TRMM project and uses the current generation of algorithms for the entire time period. This reprocessing was finished for the TRMM era in late 2018, and is considered by NOAA to be of higher quality than the older CMORPH dataset (now referred to as v0.x) (Xie et al. 2017). The reprocessed v1.0 dataset, which includes a bias correction, is the CMORPH dataset used in this paper. This data is in the public domain and is available to download at ftp://ftp.cpc.ncep.noaa.gov/precip/global_CMORPH/30min_8km.

### Rain gauge data

We use rain gauge data from two different geographic regions. The first is an individual rain gauge used in the Salinity Processes in the Upper Ocean Regional Study (SPURS-II) project, located in the Pacific Ocean near 10°N 125°W which recorded accumulation every minute for 1 year (Farrar and Plueddemann 2019; Farrar 2020). We will compare irregular sub-daily fluctuations computed via the Covey et al. (2018) analysis applied to the SPURS-II 1-min data with irregular sub-daily fluctuations computed from SPURS-II data smoothed to 1 h. We also use 4 years of hourly data from a cluster of seven land-based rain gauges in the National Oceanic and Atmospheric Administration (NOAA)'s Local Climatological Dataset. The cluster is made up of rain gauges from major US cities (Charleston, Jacksonville, Melbourne, Miami, Norfolk, Savannah, and Wilmington) between 25°N and 38°N near the Atlantic Coast.

### ECMWF ERA5 reanalysis

As an intermediary between observational products and “pure" numerical models, we analyze a reanalysis product, the ECMWF version 5 (ERA5; European Centre for Medium-Range Weather Forecasts 2018). The ERA5 output is provided hourly on a 0.25° grid. As of June 2019, the dataset goes back to 1979, but we choose a 5-year period (1998–2002) that begins on the same date as TRMM in 1998. ERA5 shares an atmospheric model base (Integrated Forecast System–IFS) with the EC-Earth runs (described below, in Sect. 2.6), thus providing a useful way to assess the effect of data assimilation. Unlike the other global models considered in this study, the underlying model in the ECMWF ERA5 reanalysis is not coupled to a dynamical ocean model. Instead, ERA5 uses a prescribed sea surface temperature.

### US Navy ESPC

The US Navy Earth System Prediction Capability (ESPC) coupled atmosphere–ocean model (Barton et al. 2021) was developed by the Naval Research Laboratory for operational weather and ocean forecasting. It incorporates data assimilation in both the atmosphere and ocean, providing a second point of entry into the effects of data assimilation, although in this case we do not have a non-assimilative twin to compare with. The ocean component is the HYbrid Coordinate Ocean Model (HYCOM), with a nominal grid spacing of 0.04°, and the atmosphere grid spacing is 19 km. Precipitation output is placed on a 0.5° grid. For this study we used 1 year of precipitation output at 3 h intervals. The Navy ESPC model is the only weather forecast model used in this study.

The Navy ESPC runs use a modified Kain-Fritsch convection scheme (Kain and Fritsch 1990, 1993) which includes a closure for Kain-Fritsch dynamically forced modes. At every time step, the scheme is called, and the cloud base mass flux is adjusted as in the schemes of Emanuel (1991) and Emanuel and Zivkovic-Rothman (1999). Boundary layer plume effects are partially represented with a convective trigger formulation (Ridout and Reynolds 1998).

### EC-earth

We use the data from two high-resolution coupled ocean–atmosphere simulations performed with version 3.2 of the global coupled climate model EC-Earth (Hazeleger et al. 2010, 2012). The atmospheric component of EC-Earth is the Integrated Forecast System (IFS) of the European Centre for Medium Range Weather Forecasts (ECMWF). Based on cycle 36r4 of IFS, it is used at T255 and T1279 horizontal resolutions (~ 80 and 16 km, respectively), using a reduced Gauss-grid. The model has 91 vertical levels, with 50 lying above 200 hPa. The model top is at 0.01 hPa. The ocean component is the Nucleus for European Modelling of the Ocean (NEMO, Madec 2008). It uses a tri-polar grid with poles over northern North America, Siberia and Antarctica with a nominal grid spacing of about 1/12th degree (the so-called ORCA12-configuration, ~ 9 km) and 75 vertical z-coordinate levels. The version of NEMO is 3.6 and includes the *Louvain la Neuve* sea-ice model version 3 (LIM3, Vancoppenolle et al. 2012), which is a dynamic-thermodynamic sea-ice model (Note: EC-Earth3.2 uses LIM3 with only one sea ice category. Both the ocean/sea-ice and atmosphere components are run with a 6-min time step.) The atmosphere and ocean/sea ice parts are coupled through the OASIS-MCT (Ocean, Atmosphere, Sea Ice, Soil) coupler (Craig et al. 2017) every 12 min. These simulations were performed in the context of the WP4 of the PRIMAVERA H2020 project. The EC-Earth simulations with low- and high-resolution atmospheric components are henceforth referred to as "EC-Earth low" and "EC-Earth high", respectively. Both experiments have an output interval of 6 h and an output duration of 1 year.

The EC-Earth convective parameterizations (Bechtold et al. 2008) feature an improved accounting of entrainment in plumes that undergo deep convection. The scheme is more sensitive to environmental moisture, and improves precipitation patterns in the tropics as well as the mid-latitude atmospheric circulation.

### NOAA GFDL

All of the Geophysical Fluid Dynamics Laboratory (GFDL) simulations (Griffies et al. 2015) have a relatively coarse horizontal atmospheric grid spacing of 0.5°, but differ in the ocean grid spacing: 1° (CM2-1deg), 0.25° (CM2.5), and 0.1° (CM2.6). This model hierarchy is valuable for ascertaining the effects of ocean model grid spacing on precipitation statistics. The GFDL simulations were run over more than 100 years with fixed atmospheric greenhouse gas concentrations corresponding to 1990. Model results from years 111–130 are analyzed in this paper using 1-day mean fields.

The GFDL climate models use the AM2 atmospheric physics as documented in Anderson et al. (2004). As noted there, the atmospheric convective parameterization makes use of the Relaxed Arakawa–Schubert scheme of Moorthi and Suarez (1992); detrainment of cloud liquid, ice, and fraction from convective updrafts into stratiform clouds; a lower bound imposed on lateral entrainment rates for deep convective updrafts (Tokioka et al. 1988); and convective momentum transport represented by vertical diffusion proportional to the cumulus mass flux.

### CESM/CCSM

Model runs were performed at National Center for Atmospheric Research (NCAR; Small et al. 2014) and Rosenstiel School of Marine and Atmospheric Sciences (RSMAS; Kirtman et al. 2012) for three resolutions of the Community Earth System Model (CESM) base and for two resolutions of the Community Climate System Model (CCSM) base.

For the highest-resolution CESM model run (henceforth "CESM high"), a 0.25° atmosphere (on the spectral element CAM5-se-NE120 grid; Park et al. 2014) was coupled to a 0.1° ocean (the Parallel Ocean Program model, version 2-POP2; Smith et al. 2010). Community Ice Code version 4 (Hunke and Lipscomb 2008), Community Land Model version 4 (Lawrence et al. 2011) and the CESM Coupler 7 with the Large and Yeager (2009) air-sea flux routine were also included. We employ “present-day” (year 2000) greenhouse gas conditions. In the mixed-resolution model runs (henceforth "CESM mixed") the same atmospheric component was coupled to a lower-resolution (1° grid spacing) ocean, in which eddies are parameterized via the Gent and McWilliams (1990) scheme. The third model run (henceforth "CESM low") done at NCAR employs a grid spacing of 1° for both the atmospheric and oceanic components. We analyze 20-year output subsets of daily-averaged precipitation fields from all considered CESM simulations.

The CESM shallow convection scheme (Park and Bretherton 2009) carries out vertical transport via ensemble-mean updraft plumes. The deep convection scheme (Neale et al. 2008) has a closure based upon dilute convective available potential energy (CAPE) along with vertical transport of horizontal momentum (Richter and Rasch 2008).

We also use a low-resolution CCSM model run ("CCSM low") from RSMAS (LRC08), in which daily-averaged precipitation values are saved every 2 days over a period of 14 years from a 0.5°- and 1°-atmosphere and 1° ocean. We are able to use only the first 8 years of output for this paper due to a discontinuity present in the data starting shortly afterwards. RSMAS also ran a higher resolution ("CCSM high") ocean version of CCSM (HRC10) with a 0.5° atmosphere and 0.1° ocean; however, it collected only monthly-averaged data and was therefore omitted from this study. A CCSM high spectrum is plotted among the spectra in Fig. 5, but is not shown in many of the other analyses in this paper due to its low-frequency output.

### GEOS/ECCO

The GEOS/ECCO coupled model employs the Goddard Earth Observing System (GEOS) infrastructure and atmospheric model coupled to the Massachusetts Institute of Technology general circulation ocean model (MITgcm) and the Community Ice Code version 4 (Hunke and Lipscomb 2008). A description of the GEOS atmospheric model and the atmosphere–ocean coupling is found in Molod et al (2020). The atmospheric horizontal grid is a cubed sphere (Putman and Lin 2007) discretization and the vertical grid is a hybrid sigma-pressure coordinate with 72 levels. The simulation used the Relaxed Arakawa Schubert (RAS) cumulus parameterization (Moorthi and Suarez 1992) with a stochastic Tokioka limit (Molod et al. 2015), and the two-moment cloud microphysics of Barahona et al. (2014). MITgcm has a finite volume dynamical core (Marshall et al. 1997). It has a nonlinear free-surface and real freshwater flux (Adcroft and Campin 2004) and a nonlocal K-profile parameterization scheme for mixing (Large, et al. 1994). The MITgcm horizontal grid type is the so-called "Lat-Lon-Cap'' (Forget et al. 2015), and the vertical grid type is the z* height coordinate (Adcroft and Campin 2004) with 90 vertical levels.

The "GEOS low" (c720-llc1080) model has 1/8° atmospheric and 1/12° oceanic components. The "GEOS high" (c1440-llc2160) model has 1/16° atmospheric and 1/24° oceanic components. Temporal resolution for each model’s output is 1 h. As of August 2020, when the model output was downloaded, GEOS low contained 1 year of output, while GEOS high contained only 84 days (from April 13 to July 5 of 2012). Due to this short timespan, and the fact that GEOS high records precipitation rate at snapshots 1 h apart while GEOS low records 1-h accumulation of precipitation, comparisons of the two GEOS simulations are challenging. Outputting accumulation is equivalent to outputting an average, which avoids the problem of aliasing effects at the highest frequencies.

### Table of datasets

A comparison of the main features of each dataset mentioned in Sect. 2 is shown in Table 1.

**Table 1:** List of models and datasets we analyze in this paper

Name	Type	Atmospheric model grid spacing	Ocean model grid spacing	Output grid	Output period (for version used)	Duration (for the portion that we use)	Start date	End date
CMORPH	Satellite dataset	n/a	n/a	0.25°	3 h	17 years	1998-01-01	2014-12-31
TRMM	Satellite dataset	n/a	n/a	0.25°	3 h	17 years	1998-01-01	2014-12-31
SPURS-II rain gauge	Rain gauge	n/a	n/a	n/a (1 gauge)	1 min and 1 h versions both used	1 year	2016-08-24	2017-08-23
NOAA rain gauge cluster	Rain gauges	n/a	n/a	n/a (7 gauges)	1 h	4 years	2016-01-01	2019-12-31
ERA5	Reanalysis	0.25°	n/a	0.25°	1 h	5 years	1998-01-01	2002-12-31
US Navy ESPC	Coupled atmosphere–ocean forecast model	19 km	0.04°	0.5°	3 h	1 year	2018-09-01	2019-08-31
EC-Earth high	Coupled ocean–atmosphere model	≈ 15 km (T1279 grid)	0.083°	≈ 0.14°	6 h	1 year	1990-01-01	1990-12-31
EC-Earth low	Coupled ocean–atmosphere model	≈ 60 km (T255 grid)	0.083°	≈ 0.70°	6 h	1 year	1990-01-01	1990-12-31
CM2.6	Coupled ocean–atmosphere model	0.5°	0.1°	≈ 0.5°	1 day	20 years	111-01-01	130–12-31
CM2.5	Coupled ocean–atmosphere model	0.5°	0.25°	≈ 0.5°	1 day	20 years	111-01-01	130-12-31
CM2-1deg	Coupled ocean–atmosphere model	0.5°	1°	≈ 0.5°	1 day	20 years	111-01-01	130-12-31
CESM high	Coupled ocean–atmosphere model	0.25°	0.1°	≈ 0.25°	1 day	20 years	61-01-01	80-12-31
CESM mixed	Coupled ocean–atmosphere model	0.25°	1°	≈ 0.25°	1 day	20 years	01-01-01	20-12-31
CESM low	Coupled ocean–atmosphere model	1°	1°	≈ 1°	1 day	20 years	01-01-01	20-12-31
CCSM high	Coupled ocean–atmosphere model	0.5°	0.1°	≈0.5°	2 days	8 years	256-12-03	264-12-01
CCSM low	Coupled ocean–atmosphere model	0.5°	1°	≈ 0.5°	1 month	30 years	01-01	30-12
GEOS high	Coupled ocean–atmosphere model	0.0625°	0.042°	0.0625°	1 h	84 days	2012-04-13	2012-07-05
GEOS low	Coupled ocean–atmosphere model	0.125°	0.083°	0.125°	1 h	1 year	2012-02-08	2013-02-06

## Example time series

**Fig. 1:**
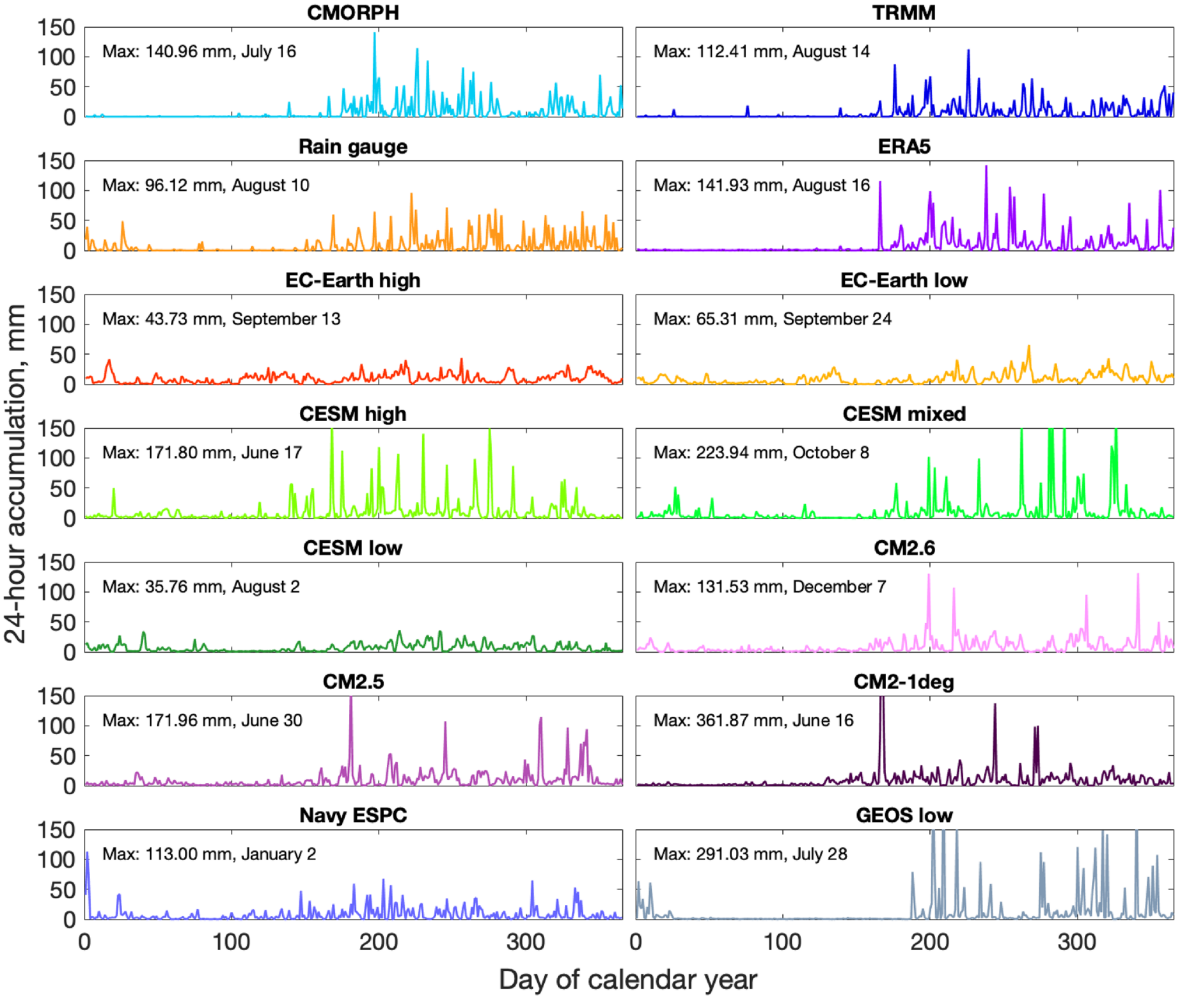
Precipitation time series at the SPURS-II rain gauge location (10°N 125°W). Where available, 1 year of continuous daily data is shown; the subplots for the rain gauge, Navy ESPC, and GEOS low time series have discontinuities where the start and end dates of output (Table 1) are displayed adjacent to each other. The y-axis shows 24-h precipitation accumulation for each day in the year shown. For each subplot, maximum values over the 1 year time series are given in text

To provide context for the analyses of irregular sub-daily fluctuations, frequency spectra, and cumulative distribution functions, we first showcase 1-year time series of daily accumulation amounts from most of the datasets we analyze at the SPURS-II location. All of the time series plotted in Fig. 1 contain daily samples over at least 1 year. The raw time series highlight different behaviors across the models and datasets analyzed in this work. For instance, the drizzle effect is clearly seen; the observational group of datasets in the top panel contain many days with zero rainfall accumulation, whereas the model datasets (for instance the GFDL CM grouping) conversely have almost no days with zero accumulation. The extent of the drizzle effect appears to vary more across different model classes than it does based on grid spacing within model groupings. The SPURS-II site is in a deep-convection region (the eastern Pacific Inter-tropical Convergence Zone) and has some of the highest precipitation rates on the globe. Some caution is required in trying to interpret the differences in the various precipitation time series in Fig. 1, because the general trends seen at this location are not always consistent with behaviors seen at other locations (as shown in Fig. 10, to be discussed later).

## Application of Covey et al. (2018) analysis of irregular sub-daily fluctuations

Our first analysis focuses on irregular sub-daily fluctuations, following Covey et al. (2018), who developed the technique and applied it to satellite datasets and coarser grid spacing (1°) CESM simulations. Covey et al. (2018) demonstrated that precipitation variance of CMORPH and TRMM is dominated by irregular sub-daily fluctuations, rather than daily means or the mean diurnal cycle. Covey et al. (2018) also showed that irregular sub-daily fluctuations were much too weak in the 1° CESM simulations relative to CMORPH and TRMM (see their Fig. 2a). Here we apply the Covey et al. (2018) technique to rain gauge data and to those higher-resolution simulations that have subdaily precipitation outputs.

**Fig. 2:**
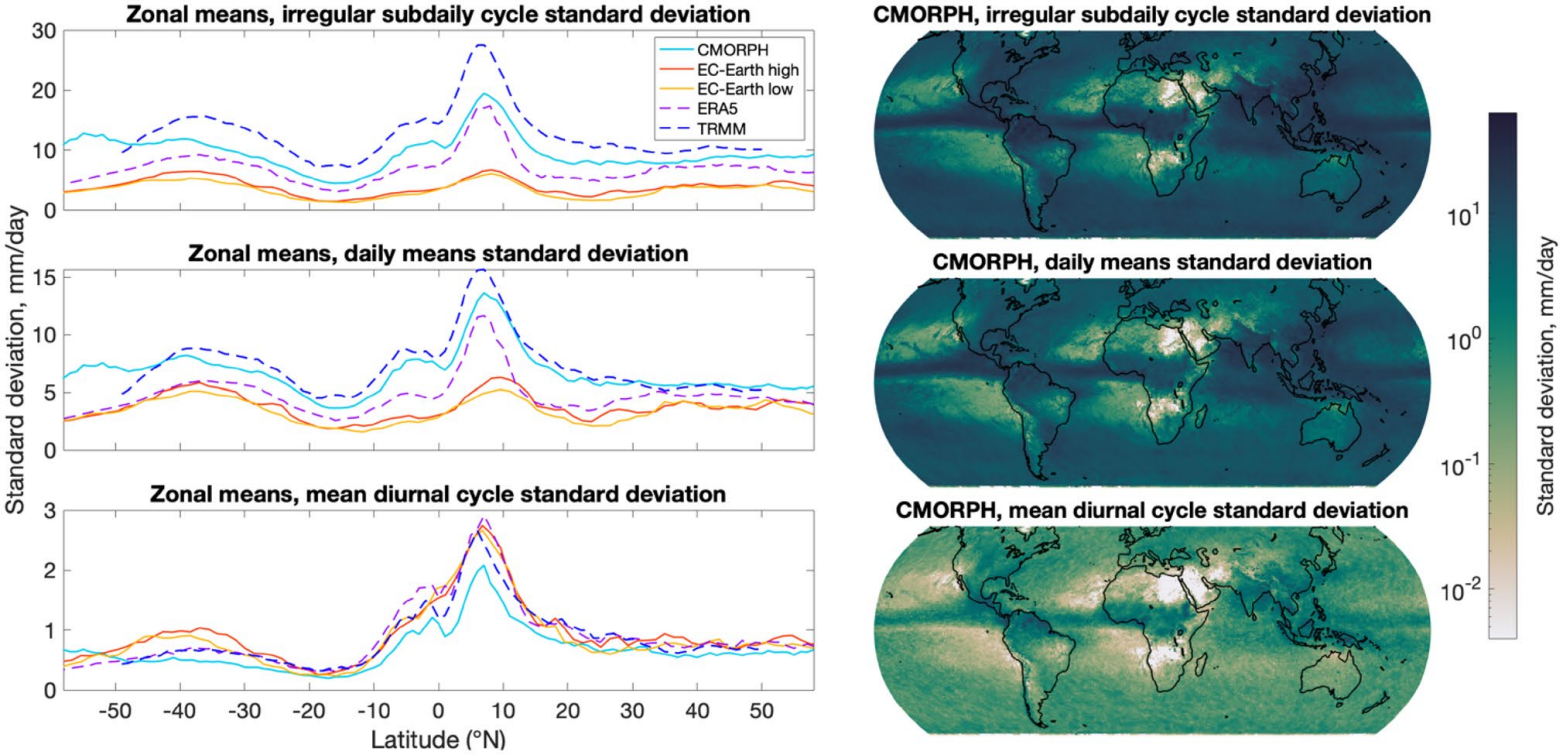
Standard deviations for (top row) irregular sub-daily fluctuations, computed as a residual, (middle row) daily means, and (bottom row) the mean diurnal cycle. Analysis follows that of Covey et al. (2018). Units of standard deviation are mm/day. Right-hand side subplots are global maps made from 1° by 1° subsamples of 3-hourly CMORPH data, while the left-hand side subplots display zonal means of CMORPH, TRMM, ERA5, EC-Earth high, and EC-Earth low. Note the different y-axis scales amongst the left-hand side subplots

Following Covey et al. (2018), we perform analysis on 1 month of the year; where possible, the same month sampled over several years. Covey et al. (2018) focused on the month of July. Due to the limited availability of output from GEOS high, we focus instead on June here. (However, June is a dry month in the SPURS-II rain gauge record, so we instead use November for that site.) We write the time series of precipitation at a grid point as *X*_*i,n*_, where *i* = 1, 2, *…, D* is an index denoting time during the day (*D* is 8 for 3-hourly data, and 24 for hourly data) while *n* = 1, 2, *…, N* is an index denoting the day of the month. For the 1-min SPURS-II rain gauge data, *D* = 24 (the 1-min data is averaged over hourly intervals). We define the following means:

- the mean diurnal cycle $\overline{{X }_{i}^{dc}}=\frac{1}{N}\sum_{n}{X}_{i, n},i=1, 2, \dots , D$
- the daily mean $\overline{{X }_{n}^{dm}}=\frac{1}{D}\sum_{i}{X}_{i, n}, n=1, 2, \dots , N$
- the overall monthly mean $\overline{{X }^{all}}=\left(\frac{1}{D}\right)\left(\frac{1}{N}\right)\sum_{i, n}{X}_{i, n}=\frac{1}{N}\sum_{n}\overline{{X }_{n}^{dm}}=\frac{1}{D}\sum_{i}\overline{{X }_{i}^{dc}}$

The associated variances are given by:

- variance associated with the mean diurnal cycle ${\sigma }_{mdc}^{2}$ =$\frac{1}{D}\sum_{i}{\left(\overline{{X }_{i}^{dc}}-\overline{{X }^{all}}\right)}^{2}$
- variance associated with the daily mean ${\sigma }_{dm}^{2}$ =$\frac{1}{N}\sum_{n}{\left(\overline{{X }_{n}^{dm}}-\overline{{X }^{all}}\right)}^{2}$
- overall variance $${\sigma }_{all}^{2}$$ =$$\frac{1}{D}$$ $$\frac{1}{N}\sum_{i, n}{\left({X}_{i, n}-\overline{{X }^{all}}\right)}^{2}.$$

The overall variance ${\sigma }_{all}^{2}$ is written as the sum of three components:

$${\sigma }_{all}^{2}={\sigma }_{mdc}^{2}+{\sigma }_{dm}^{2}+{\sigma }_{isd}^{2}$$

where the residual ${\sigma }_{isd}^{2}$ is associated with irregular sub-daily fluctuations.

Global maps of the standard deviation (square root of the variance) of irregular sub-daily fluctuations, daily means, and mean diurnal cycle, computed from 17 Junes of the CMORPH 3-hourly product used here (left-hand subplots of Fig. 2), illustrate similar patterns as in Figure 1 of Covey et al. (2018), who used 16 Julys of an hourly CMORPH product. All three components show the least amount of variance over dry regions in Africa (Sahara Desert, Namib/Botswana Deserts) and over the eastern subtropical gyres in the ocean. Zonal means of CMORPH (right-hand subplots) are of similar magnitude as the zonal means displayed in Covey et al. (2018). Along with the zonal means of CMORPH in Fig. 2, we display zonal means of TRMM, ERA5, EC-Earth high, and EC-Earth low. The CMORPH and TRMM data used for the analysis are subsampled from points 1° apart, not regridded to simulate 1° grid spacing. As in Covey et al. (2018), the TRMM zonal means are similar to the CMORPH zonal means. Our analysis demonstrates that the zonal mean standard deviations of irregular sub-daily fluctuations and daily mean precipitation are generally higher in the data-assimilative ERA5 reanalysis than in the free-running EC-Earth models, such that ERA5 lies closer to CMORPH, while the mean diurnal cycle does not show a significant change between ERA5 and EC-Earth, which both lie close to CMORPH results. The ERA5 reanalysis employs the same dynamical-core atmospheric model as EC, but we have a longer time series for ERA5 (5 years) than for EC-Earth (1 year). The data assimilation employed in ERA5 is another critical difference between it and EC-Earth.

**Fig. 3:**
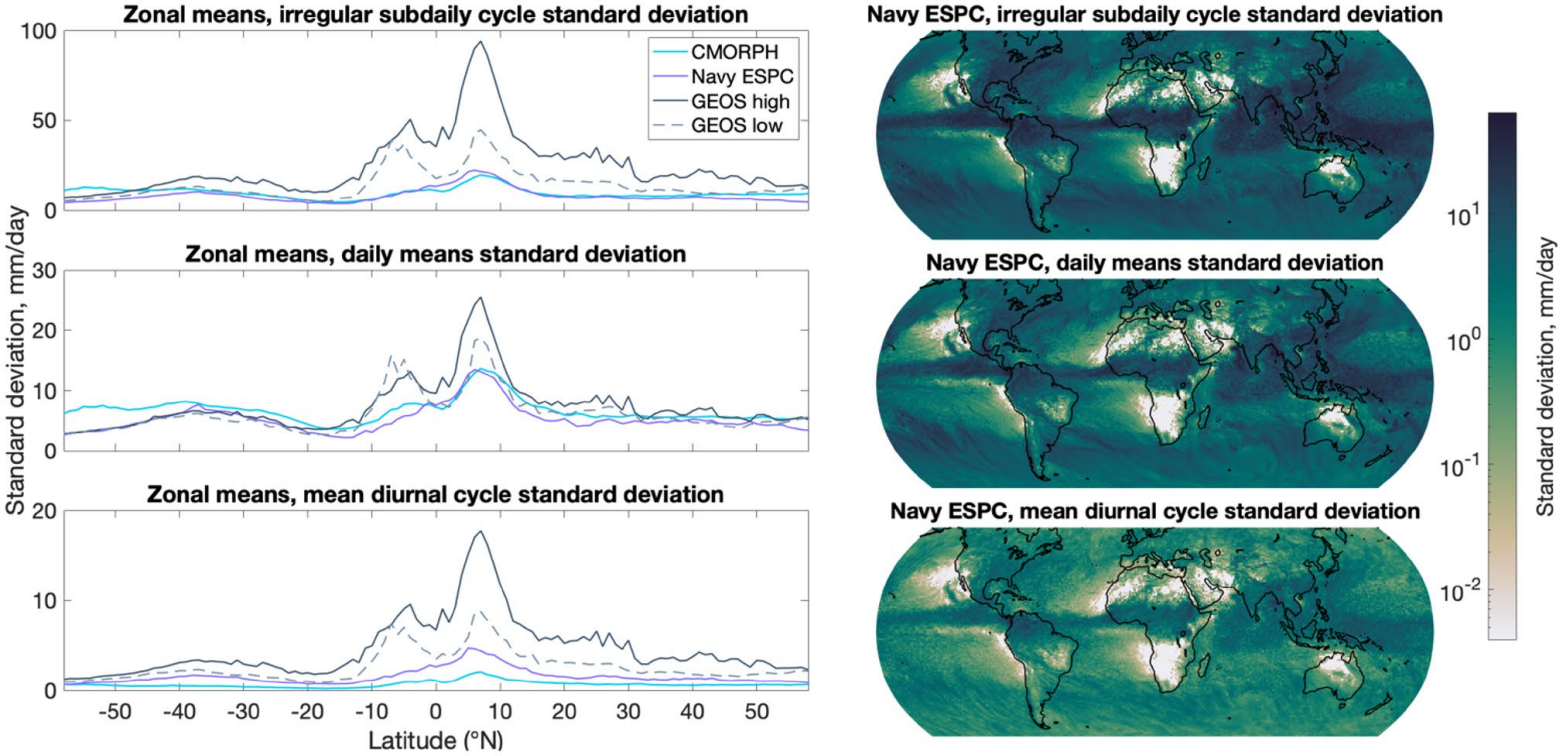
As in Fig. 2, except that right-hand side subplots are global maps made from 1° by 1° subsamples of 3-hourly Navy ESPC data, while the left-hand side subplots display zonal means of CMORPH, Navy ESPC, GEOS high, and GEOS low. The scales on the left-hand subplots differ from each other and from the scales on the left-hand subplots of Fig. 2

Global maps of the standard deviation of irregular sub-daily fluctuations, daily means, and mean diurnal cycle from Navy ESPC are displayed in the left-hand side subplots of Fig. 3, while zonal means of the Navy ESPC, GEOS high, and GEOS low are displayed in the right-hand side subplots. In contrast to the EC-Earth results in Fig. 2 and the 1° CESM results in Covey et al. (2018), which display weak irregular sub-daily fluctuations, the zonal mean of the irregular sub-daily fluctuations in the Navy ESPC model lies close to CMORPH, and the GEOS low and GEOS high zonal means are significantly higher. The Navy ESPC daily means also lie close to CMORPH, while the GEOS models again lie too high. The Navy ESPC and both GEOS zonal means of the diurnal cycle are all too high relative to CMORPH. As seen in “Appendix” (Fig. 11), zonal averages of irregular sub-daily fluctuations computed from individual years of CMORPH observations are generally less than zonal averages computed from the entire 1998–2014 CMORPH observational dataset, but the differences are generally smaller than differences in zonal averages of the GEOS results versus the CMORPH results. Thus, the shorter 1-year duration of the GEOS results is not the reason for the discrepancy between GEOS and CMORPH. Analysis with cumulative distribution functions and observed time series (Figs. 1 and 6) have shown both GEOS/ECCO model outputs to have a higher fraction of precipitation derived from major events rather than drizzle; these major events may underlie the high GEOS/ECCO irregular sub-daily fluctuation values. Another possible cause for the discrepancy between CMORPH and GEOS/ECCO values is the respective 3-hourly vs. hourly sampling. The analysis performed in Covey’s paper showed greater irregular sub-daily variance for an hourly version of CMORPH than for 3-hourly TRMM. In this paper we use the 3-hourly version of CMORPH to align with the sampling intervals of TRMM output. We note as well that the GEOS/ECCO models are the highest resolution atmospheric models in this study, which may be related to their high irregular sub-daily fluctuation values relative to CMORPH. Another factor that may be relevant to the large high-frequency precipitation variance in GEOS/ECCO is the coupling frequency. In GEOS high simulations, information is exchanged between the ocean and atmosphere every 45 s, while in GEOS low it is exchanged every 120 s. In contrast, ERA5, for example, uses daily SST, while the GFDL CM2.5/2.6 simulations couple every 1200/3600 s, respectively. We will see again in Fig. 8 a tendency for the GEOS models to have more high-frequency variance than is seen in other models or even sometimes in observations. The Navy ESPC models also have a high resolution relative to most of the other models used in this study, and have the added advantage of data assimilation.

“Appendix” includes results of the Covey et al. (2018) analysis applied to rain gauge records and to CMORPH, Navy ESPC, GEOS low, and GEOS high output at the rain gauge locations (Fig. 12). As these are single-point rain gauges, the number of samples is relatively small and the means and variances can change significantly from year to year. The 2019 results for the NOAA Charleston rain gauge are dominated by one large event and are much larger than the results for 2016–2018. What is robust across all rain gauge results is that irregular sub-daily fluctuations dominate the overall variance, consistent with Covey et al. (2018), and that this dominance is even greater in analysis of longer records. In individual year records from the seven NOAA gauges, the irregular sub-daily fluctuations account for 74–93% of the overall variance. In analysis of the 4-year NOAA records, the irregular sub-daily fluctuations account for 95–98% of the overall variance. Application of the Covey et al. (2018) analysis to November 2016 of the 1-year SPURS-II rain gauge yields similar conclusions. Irregular sub-daily fluctuations account for 98% of the total variance in 1-min SPURS-II data, and 88% of the total variance in hourly SPURS-II data, a smoothed version of the 1-min data. The hourly SPURS-II data contains about five times less variance than the 1-min data. Another important statistic that changes between the 1-min SPURS-II data and the (smoothed) hourly SPURS-II data is the fraction of time with non-zero precipitation – 1.5% for the former versus 6.4% for the latter. These differences between the 1-min and hourly SPURS-II results further demonstrate the importance of high frequency output for temporally intermittent quantities such as precipitation. In Fig. 12, as in Fig. 11, we see a tendency for the ratio of variance in irregular sub-daily fluctuations to total variance computed from CMORPH to increase as the record length increases. We also see in Fig. 12 a tendency for this ratio to be larger in Navy ESPC and especially GEOS low/GEOS high than in CMORPH, and for the values of the ratio computed from the 1-year-or-less GEOS model records to lie closer to the values computed from rain gauges than do the values of the ratio computed from individual years of CMORPH output.

Our application of the Covey et al. (2018) analysis to higher-resolution models and to both free-running and data-assimilative models illustrates the value of both fine grid spacing and data assimilation for matching the behavior of high-frequency precipitation variance seen in observations. However, the Covey et al. (2018) analysis requires sub-daily intervals of precipitation outputs, whereas even many high-spatial-resolution models provide only daily outputs. Considering these results, it is clear that modeling centers should consider outputting hourly precipitation values over years for which high-quality precipitation observations are available for comparison. In the meantime, in order to examine the other high-spatial-resolution simulations in this paper, which do not provide sub-daily output, we turn to other tools (frequency spectra and cumulative distribution functions) and focus on slightly longer time scales (2–100 days) for the remainder of the paper.

## Frequency spectra and cumulative distribution functions

### Methods

**Fig. 4:**
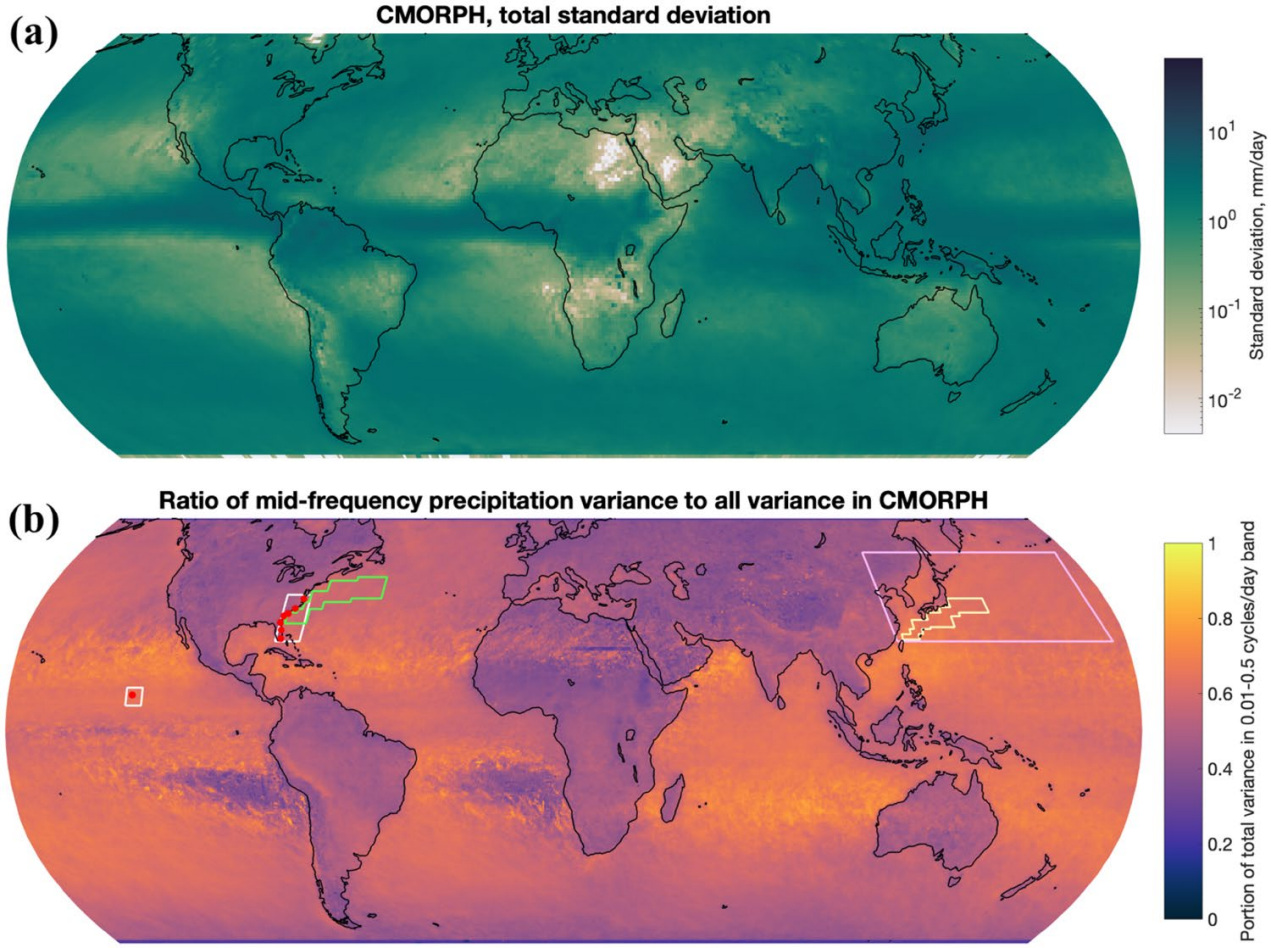
**a** Maps of the total standard deviation in CMORPH, calculated as the square root of the total variance in the CMORPH record. **b** Ratio of CMORPH precipitation variance in the 0.01–0.5 cycles/day frequency (2–100 day period) band to the total CMORPH variance. Colored (non-white) boxed areas represent regions that spectra in Fig. 9 are averaged over, red points are locations of rain gauge datasets used here, and white boxes represent areas around those rain gauges over which we averaged model results for Figs. 5, 6 and 8. The white boxes surround red dots representing the rain gauge locations themselves. Green box is the Gulf Stream region, orange box is the Kuroshio region, and pink box is the Northwest Pacific region. The Atlantic Coast region (which contains 7 rain gauges) overlaps with the Gulf Stream region, and the SPURS-II rain gauge area is located in the eastern tropical Pacific Ocean

In this section we examine precipitation variance spectra averaged over specific regions. We are largely interested in the mid-frequency (0.01–0.5 cycles/day) band (henceforth, often referred to as the “mid-frequency band”), which overlaps both the time scales of oceanic mesoscale eddies and the output time scales of the models and observational products examined here. Precipitation variance at higher frequencies will be examined briefly in the cases where output frequency allows. The total standard deviation computed from frequency spectra (Fig. 4a) resembles the standard deviation computed from the Covey et al. (2018) analysis, e.g., the square root of the sum of the squares of the maps in the left-hand side of Fig. 2. The resemblance is not total because the Covey et al. (2018) analysis in Fig. 2 is computed from 17 Junes whereas the standard deviation shown in Fig. 4a is computed from the full 17 years of CMORPH data including all months. The fraction of precipitation variance derived from CMORPH data that is in the 0.01–0.5 cycles/day band, compared to the variance in the full measurable range in that dataset of 0.000161 cycles/day-4 cycles/day, is displayed in Fig. 4b. The ratio in Fig. 4b is substantial, up to 0.9 in some locations, justifying this choice of frequency band as interesting and significant. This ratio is also generally higher in oceanic regions, further justifying the choice of band as relevant to studying the impact of oceanic mesoscale eddies on precipitation output. Later in the paper, we display both spectra averaged over many model grid points in specific regions, along with global maps of variance differences in the 0.01–0.5 cycles/day band to highlight the effects of grid spacing refinement for model grid points worldwide. Cumulative distribution functions (CDFs) of daily precipitation accumulation computed over the same regions are also shown, largely because CDFs are commonly used (e.g., United States Department of Energy 2020). Showing CDFs in conjunction with spectra is valuable because we can view both the sizes of precipitation events that occur and the amount of total precipitation associated with particular frequencies.

**Fig. 5:**
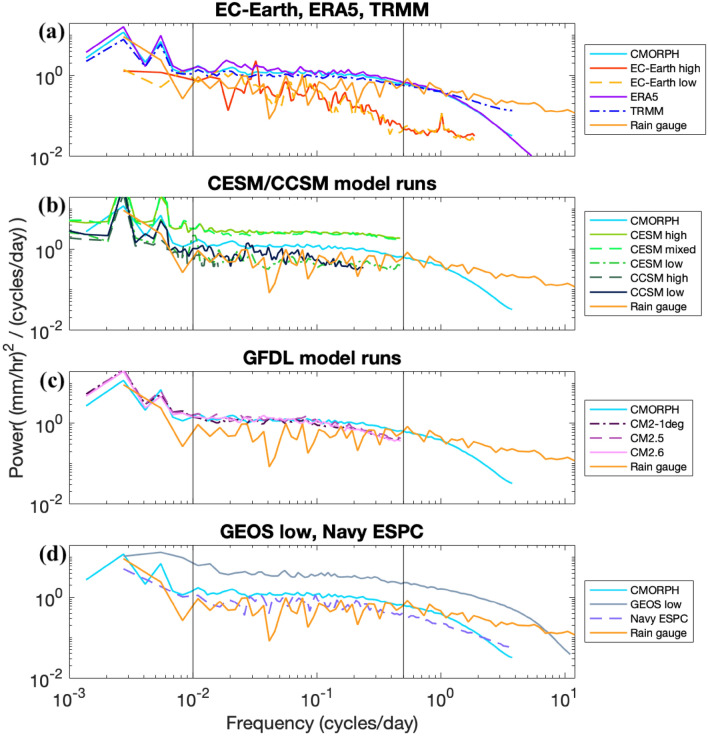
Precipitation variance spectra at the SPURS-II location (10°N 125°W). Black vertical lines denote 0.01 and 0.5 cycles/day: the edges of the mid-frequency band highlighted in this section of the paper

**Fig. 6:**
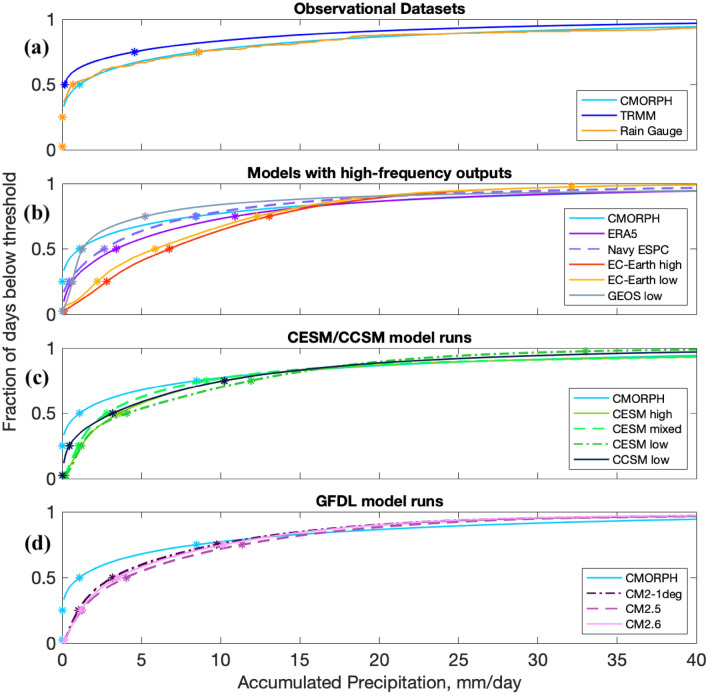
Cumulative distribution functions for precipitation accumulation at the SPURS-II location. These plots show the fraction of days that have less precipitation than the threshold along the x-axis. Asterisks depict the accumulation amounts associated with percentile thresholds: 2.5%, 25%, 50%, 75%, and 97.5%. Comparing the x-coordinates of these asterisks can illustrate the likelihood of drizzle or major precipitation events for each model or dataset

We carry out our spectral analysis in five regions across the globe, depicted by the boxes in Fig. 4b (region bounds provided in “Appendix”). Three regions – the Gulf Stream, the Kuroshio, and the Northwest Pacific—receive special focus due to the large air/sea heat fluxes within strong ocean currents (Yu and Weller 2007; Bishop et al. 2017; Small et al. 2019). The Northwest Pacific region, which encompasses the entirety of the Kuroshio region, was chosen in part because the GFDL model exhibits a large increase in variance over the larger region when the ocean model grid spacing is refined, and the effect is more visible over the larger Northwest Pacific region than it is over the Kuroshio current itself. The other two regions surround the SPURS-II rain gauge and the cluster of rain gauges along the Atlantic coast of the US.

We calculate precipitation variance by taking a Fast Fourier Transform (FFT) of a pre-processed precipitation rate time series expressed in mm/hr. Before the FFTs are computed, means and linear trends are removed from the data, and a Tukey window with a cosine fraction of 0.2 is applied. This Tukey window removes about 14% of the variance from a time series, and from spectra computed from such a time series. For time series that are longer than 2 years, we separate the time series into windows that are each 2 years long with 50% overlap, and compute the mean of the resulting spectra. Where possible, area-weighted spatial averaging is used to produce a single spectrum based on the spectra generated at every grid point within a region of interest. This further reduces the noise present in raw spectra, and allows us to view behaviors for an entire region instead of just a single point.

**Fig. 7:**
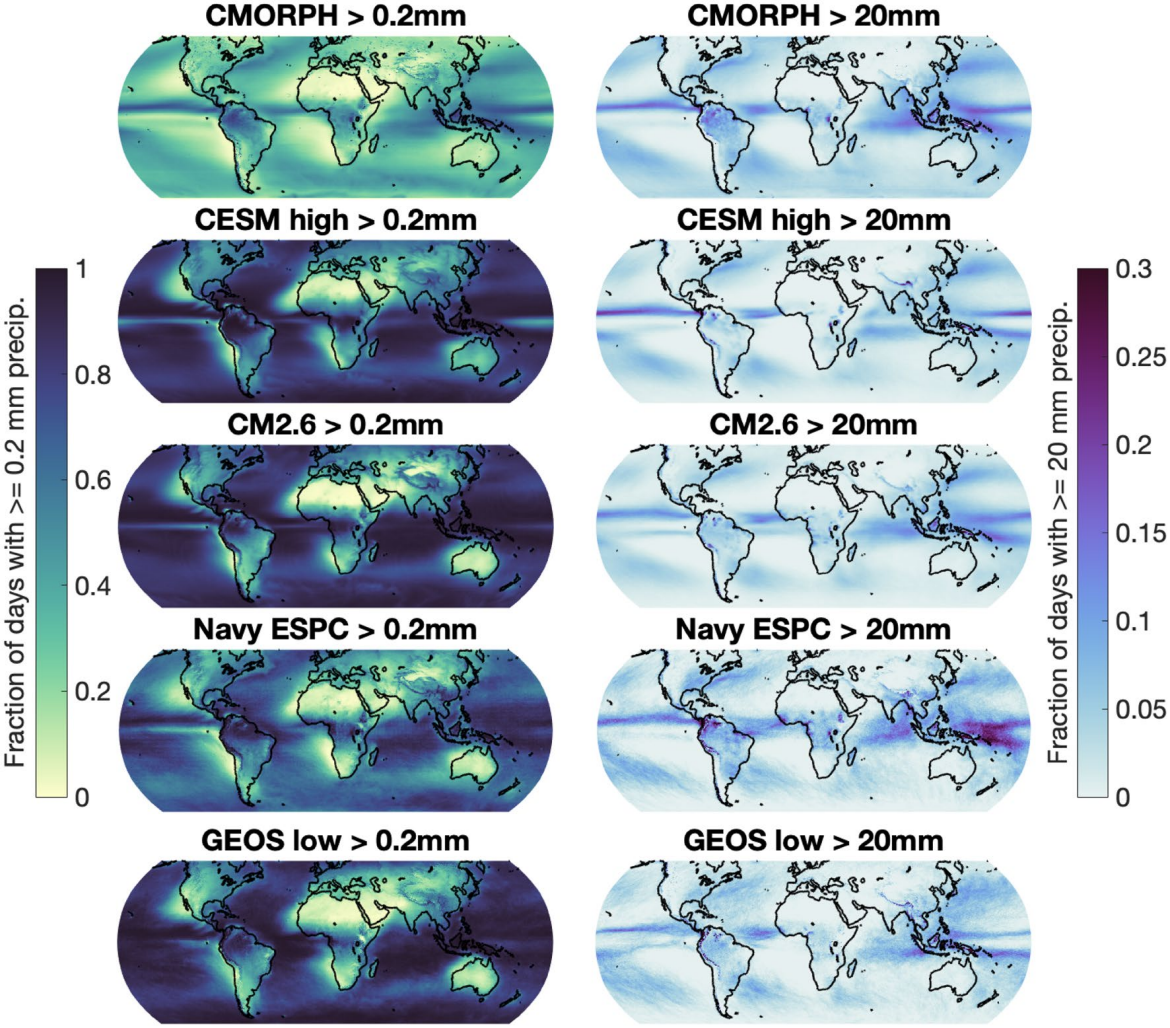
Global maps of CDFs shown in Fig. 6 at the 0.2 mm accumulation threshold (left column), and the 20 mm accumulation threshold (right column). Note the different colorbar scales between the two columns

CDFs of daily precipitation accumulation are also presented. The CDFs (Fig. 6) show the fraction of days that receive less precipitation than the specified amount of accumulation shown on the x-axis. In order to compare model groups with different temporal resolutions, all of the CDFs show accumulations over 24-h periods. The CDF plots are particularly useful for demonstrating the drizzle effect. Showing CDFs in conjunction with variance spectra is a powerful combination because we can view both the sizes of precipitation events that occur and the amount of total precipitation associated with particular frequencies. We also present an alternative view of CDFs on a global scale (Fig. 7), displaying the height of particular CDF vertical cross-sections at each sub-sampled grid point for several of the simulations.

**Fig. 8:**
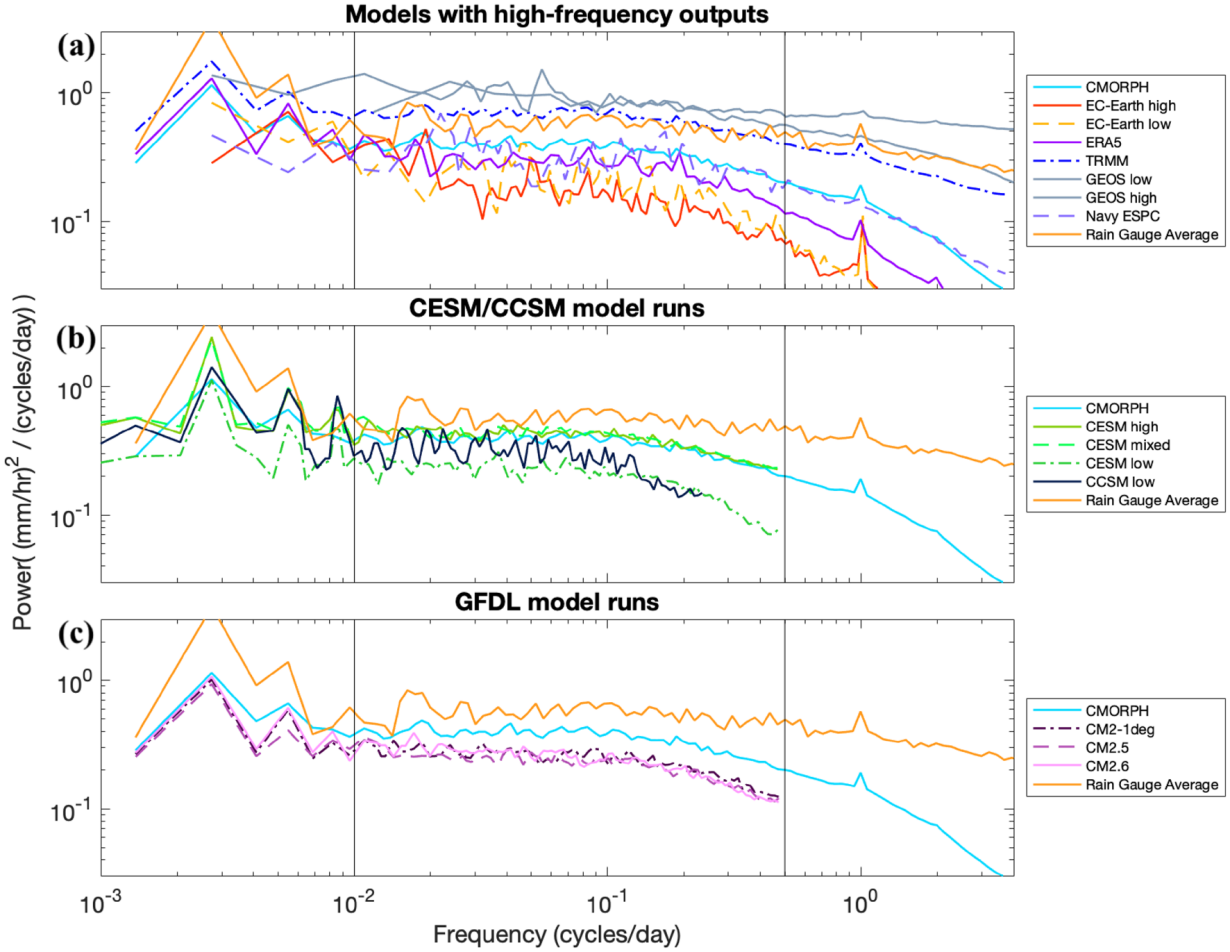
Comparison of precipitation variance spectra in rain gauges and models, as in Fig. 5, but for the Atlantic Coast region (25°–38° N, 74°–82° W), and for slightly different groupings of models (**a** combines the models shown in Fig. 5a, d, additionally showing GEOS high because seasonality of precipitation in this region is lower). Black vertical lines denote 0.01 and 0.5 cycles/day; the edges of the mid-frequency band highlighted in this section of the paper

**Fig. 9:**
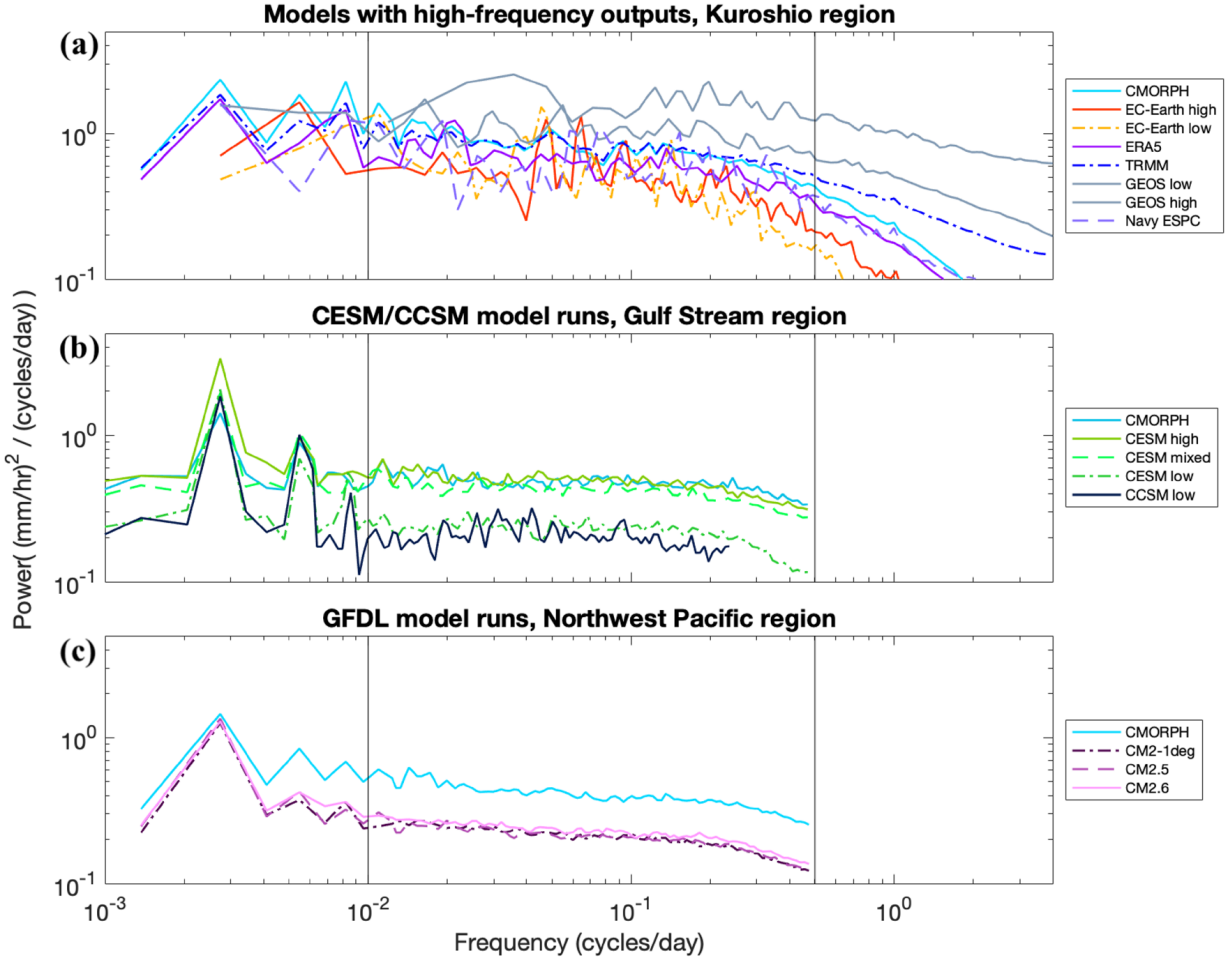
Precipitation variance spectra in **a** the Kuroshio, **b** the Gulf Stream, and **c** the Northwest Pacific. Black vertical lines denote 0.01 and 0.5 cycles/day: the edges of the mid-frequency band highlighted in this section of the paper. Subplot **a** focuses on models and datasets with high temporal resolution, **b** focuses on the CESM/CCSM model family, and **c** focuses on the GFDL model family

In Figs. 5, 6, 8, 9, simulations are organized into groups. For instance, the CESM and CCSM simulations are all grouped together, as are the GFDL CM simulations. ERA5 is grouped together with the EC-Earth simulations because they share an atmospheric core. The Navy ESPC and GEOS/ECCO models are grouped together based upon their fine grid spacings and limited record durations. Most figures include the CMORPH observational dataset for reference, and some figures also display TRMM and rain gauge observations for reference as well.

**Fig. 10:**
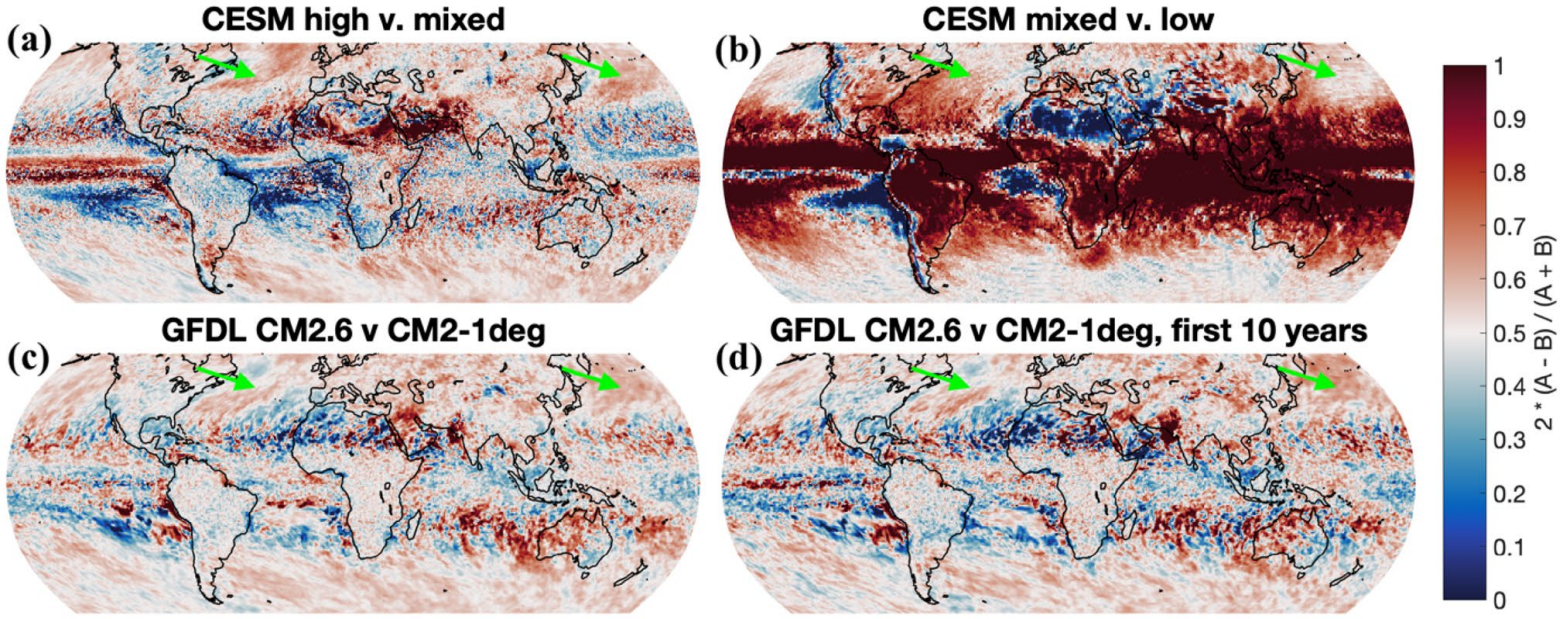
Relative differences, as given in Eq. (1), in variance sums at points spaced every 0.5° apart on the globe. Green arrows point to particular regions of interest referenced in the text. Note that the bottom two plots show the same data but for a 20-year model output duration (left) and for a 10-year model output duration (right)

**Fig. 11:**
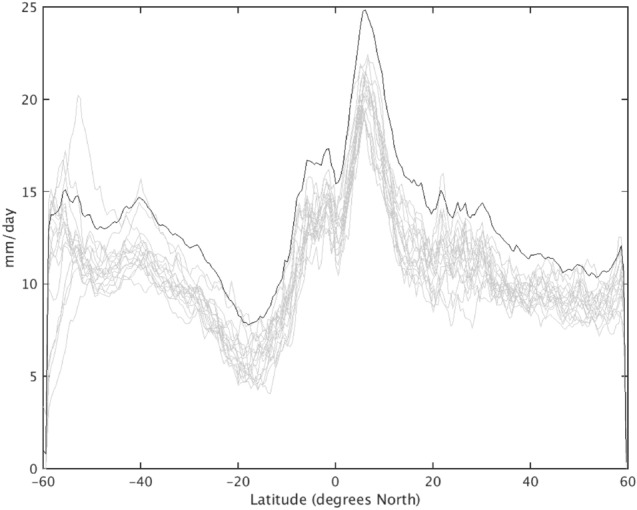
Zonal mean of the irregular sub-daily fluctuations defined in the Covey et al. (2018) analysis, computed from individual years (1998–2014) of CMORPH observations (gray curves), and from all seventeen of these years (black curve)

**Fig. 12:**
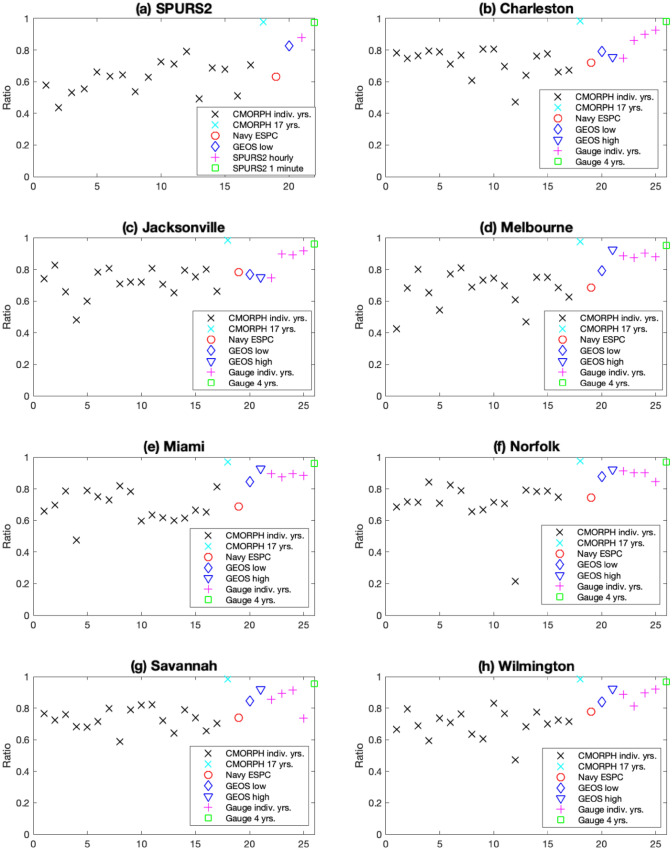
Ratio of variance in irregular sub-daily fluctuations to total variance in rain gauge records, and in CMORPH, Navy EPSC, and GEOS low/GEOS high results at the gauge locations. CMORPH results are given for 17 individual years and the full 17-year record. Results at the SPURS-II location** a** are computed from November. SPURS-II gauge results are computed from hourly and 1-min data. GEOS high results are not shown in **a** because they do not include a November. Results at the NOAA rain gauge locations **b**–**h** are computed from June. NOAA results are computed from four individual years and the full 4-year record. x-axis units are arbitrary

The global variance maps in Fig. 10 are obtained from the integral of the power spectra over the 0.01–0.5 cycles/day frequency band resolved by each model. Grid spacings of the displayed maps are roughly 0.5 degrees; for model output not stored on a Cartesian latitude–longitude grid, we plot the result for the closest available model output point to a 0.5-degree latitude–longitude grid.

### Results

**Fig. 13:**
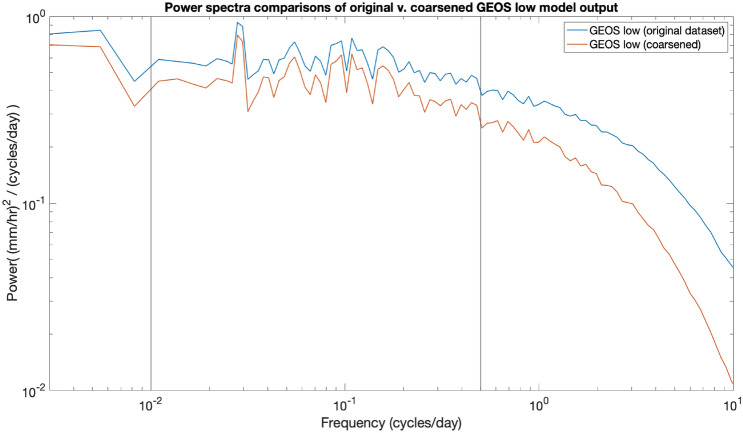
Power spectra generated from a model output (GEOS low) compared to a spatially smoothed version of the same output

We begin with precipitation rate frequency-power spectra in the SPURS-II region (Fig. 5). For some model results, annual peaks in spectra are cut off in order to magnify our focus on the mid-frequency band (0.01–0.5 cycles/day). The observational datasets and reanalyses shown in Fig. 5a tend to have more variance than the EC-Earth models at most frequencies, especially in the higher frequencies of the measured spectrum. Spatially smoothing a precipitation dataset by storing means of 16 spatial grid points at each timestamp and calculating a power spectrum from that smoothed dataset will produce less variance than the original dataset. For reference, we demonstrate the visual effect of such spatial smoothing in “Appendix” (Fig. 13). This means that we hypothetically expect to see higher variance with increasing model resolution, and the results in Fig. 5 are consistent with that hypothesis. The rain gauge spectrum is roughly consistent with the TRMM and CMORPH observations, though the rain gauge curve is noisier due to the smaller number of points in the spatial average (a single-point rain gauge compared to 400 grid points for the satellite-based datasets). The 1-min sampling of the rain gauge allows us to examine power spectra at high frequencies. Whereas the rain gauge continues to show substantial power out to 10–100 cycles/day, most of the spectra computed from models and satellite products displayed in Fig. 5 fall off steeply at frequencies somewhat greater than 1 cycle/day. Within the CESM family of models, it is evident that the change in atmospheric model grid spacing from 1.0° to 0.25° has a significant effect on variance, while the oceanic change from 1.0° to 0.1° has a modest but still noticeable effect (Fig. 5b). The high-resolution atmosphere CESM models (CESM high and CESM mixed) appear to contain more power than the other resolution CESM/CCSM model runs or the observational data. It is known that both CESM high and CESM mixed produce too much overall precipitation in this region (Small et al. 2014), and such an anomaly does increase precipitation variance in regions where it occurs; however, the greater variance occurs in other regions where annual mean precipitation is not anomalously high (which is true for most cases in our results). The CCSM runs are included in Fig. 5 (we exclude them in some other figures due to their low temporal resolution), though no strong differences are seen between the different CCSM runs within the frequency band in which they can be compared.

Within the GFDL family of models (Fig. 5c), mid-frequency variance spectra lie relatively close to CMORPH spectra. The GEOS low run (Fig. 5d) significantly overestimates the annual precipitation accumulation at this location, recording about 30% more than CMORPH (Table 2). It is suspected that this is responsible for the appearance of increased variance in that model run relative to CMORPH and the rain gauge. The GEOS high run is omitted from Fig. 5 because the output length (84 days) is not long enough to derive a useful result in regions with high seasonality of precipitation. Navy ESPC has less variance in this location compared to CMORPH, but the difference (37% less variance) is small in this region, relative to the differences that exist between some other models and CMORPH.

**Table 2:** Annual mean precipitation for both observational datasets and models at the SPURS-II location

Dataset	Annual mean (mm)
CMORPH	3117
TRMM	2894
Rain Gauge	3028
ERA5	3903
Navy ESPC	2706
EC-Earth high	3409
EC-Earth low	3232
CM2.6	3465
CM2.5	3803
CM2-1deg	3233
CESM high	4189
CESM mixed	4116
CESM low	3042
GEOS low	4101

Figure 6 displays the cumulative distribution functions (CDFs) for precipitation accumulation in the SPURS-II region. Observational datasets (shown in the top panel) have many more days with accumulated precipitation near zero compared to the models, as evidenced by the y-intercepts in Fig. 6. In the days where precipitation is recorded, the observational datasets tend to have more of it, such that annual means are not decreased in the observational datasets compared to the models. This difference in behavior between observations and models is a manifestation of the drizzle effect discussed earlier. CDFs of finer-grid-spacing models do not always closely approximate the observational-derived datasets. The EC-Earth simulations do not show the finer-grid-spacing model more closely approximating CMORPH in this region, even though that is the case in the Gulf Stream region (Fig. 15). The Navy ESPC and GEOS simulations have CDFs closer to that of CMORPH compared to the other models. Certain model groups show changes amongst their members, but there is no consistent relationship between these low- and high-resolution models. For instance, CM2.5 contains more extreme events than either the higher ocean resolution CM2.6 or the lower resolution CM2-1deg in this region (also causing an annual mean higher than the other two models as well; Table 2). The GEOS low CDF result in Fig. 6b is consistent with the high variance (Fig. 5) and accumulation (Table 2) if data located outside of the plot is taken into account; GEOS low in this region has a much higher fraction of days (2.02%) with more than 100 mm of precipitation than any of the models it is being compared against, with CMORPH having only 0.56% of days above that accumulation amount.

Global maps of vertical slices of the CDFs are displayed in Fig. 7. The drizzle effect and lack of large precipitation events are illustrated through displaying how often totals above 0.2 mm (left column) or 20 mm (right column) of daily accumulation occur at each model grid point. These global maps show the fraction of days with *at least* 0.2 or 20 mm of precipitation, whereas the CDFs in Fig. 6 show the fraction of days that have *less than* the displayed thresholds. In the maps of the left column, color shading values near 1 indicate that almost all days record an amount of precipitation large enough to be measurable. The maps in Fig. 7 vary more by model group than by grid spacing, although models involving data assimilation (Navy ESPC) and the comparatively high-resolution GEOS low model show spatial patterns closer to those in CMORPH than the other models. The more accurate CDF patterns showcase a lower fraction of days with more than a trace amount of accumulation, and a slightly greater fraction of days with high amounts of accumulation. The Navy ESPC stands out as having the lowest drizzle effect and the largest number of major precipitation events for most grid points.

Precipitation variance spectra for the Atlantic Coast region are given in Fig. 8. The rain gauge spectra represent averages over 7 spectra derived from 7 rain gauges. Differences between certain models are more visible here than in Fig. 5; the ERA5 reanalysis has more variance than the EC-Earth models, and is closer to the observational data in Fig. 8 than it is in Fig. 5 (representing contrasting results in different focus regions). Over a wide range of frequencies, the GEOS models have slightly more variance than TRMM and the average of the rain gauge cluster, with the rain gauge datasets having more variance than CMORPH or Navy ESPC in this region. The Navy ESPC spectra follow the CMORPH spectra more closely than in Fig. 5. At frequencies higher than 1 cycle/day, there is a suggestion that GEOS high retains a shallower slope than does GEOS low, in closer accordance with the rain gauge spectra. Other trends remain mostly consistent with the SPURS-II region; EC-Earth does not show a variance increase with increasing atmospheric model resolution here (although it does show one in other regions of interest), the increase in atmospheric model resolution in CESM yields a more significant change than does increasing oceanic model resolution, and the GFDL models do not show an increase in variance with increasing oceanic model resolution in this region.

Spectra averaged across the three regions that include western boundary currents (Kuroshio, Gulf Stream, and Northwest Pacific) emphasize precipitation behavior influenced by ocean mesoscale dynamics (Fig. 9). In these regions, we are particularly interested in the effects of increased ocean model resolution, which energizes ocean mesoscale eddies in the western boundary currents. We anticipate that the resulting differences in sea surface temperature from an increase in ocean model resolution will also increase the variance of precipitation (e.g., Frenger et al. 2013; Siqueira et al. 2021). We display each model grouping in a different region, based on the focus region in which they showed the strongest trends. Some of the same behaviors displayed in earlier plots are found here. The Navy ESPC spectrum closely follows CMORPH. The GEOS models, especially GEOS high, display more precipitation variance at high frequencies than the satellite products and the other models, suggesting that high spatial resolution increases high-frequency precipitation variance. Increasingly refined grid spacing in the atmospheric model increases the magnitude of CESM spectra substantially over a wide range of frequencies, while refining ocean model grid spacing makes for a slight increase in the CESM spectra in the case where the atmospheric model also has a more refined grid spacing. The GFDL models, with their coarser atmospheric model grid spacing, do not match the CMORPH spectra as well as the CESM spectra do, and a smaller increase is seen in the GFDL spectra when ocean model grid spacing is refined.

Table 3 displays the integral of the variance in the mid-frequency band (0.01–0.5 cycles/day) from most of the models shown in Fig. 9, along with boundaries for the 95% confidence intervals of each sum. Table 3 confirms some of the trends noted earlier; the increase in variance with increasing atmospheric model resolution in EC-Earth, the increase in variance with increased atmospheric model resolution in CESM, with a slight increase as ocean model resolution increases, and the slight increase in variance with increasingly refined ocean model grid spacing within the GFDL family.

**Table 3:** Integral of precipitation variance/spectral density between 0.01 and 0.5 cycles/day for certain spectra displayed in Fig. 9a–c

Model/dataset	Spectral density integral (mm/h)^2^	95% confidence interval width	Region
CMORPH	0.3170	± 3.6%	Kuroshio
EC-Earth low	0.1729	± 14.8%	Kuroshio
EC-Earth high	0.1986	± 14.8%	Kuroshio
ERA5	0.2526	± 6.6%	Kuroshio
Navy ESPC	0.2233	± 14.8%	Kuroshio
CMORPH	0.2092	± 3.5%	Gulf Stream
CESM high	0.2003	± 3.3%	Gulf Stream
CESM mixed	0.1745	± 3.3%	Gulf Stream
CESM low	0.0904	± 3.3%	Gulf Stream
CMORPH	0.1669	± 3.6%	Northwest Pacific
CM2-1deg	0.0841	± 3.3%	Northwest Pacific
CM2.5	0.0847	± 3.2%	Northwest Pacific
CM2.6	0.0921	± 3.2%	Northwest Pacific

In Fig. 10, we present the spatial distribution of variance differences between three model pairs on a global map. The variance spectra from the three model pairs are summed across the mid-frequency band (0.01–0.5 cycles/day) and we display the relative difference R:

1 $$R = 2*\left(A-B\right) / (A+B)$$

*A* is the sum in the higher resolution model and *B* is the sum in the lower resolution model. Figure 10 shows an alternative visualization to some of the same trends displayed in earlier figures. Increasing the ocean resolution from CESM mixed to CESM high displays more variance increase over the path of the Gulf Stream (where the resolving of ocean mesoscale eddies will have a larger effect) than other nearby oceanic regions. This appears to a lesser extent over the Kuroshio current as well. Increasing the atmospheric resolution from CESM low to CESM mixed displays variance increases at most model grid points, but this effect is stronger over the tropics, as well as on the west side of temperate oceans (near the currents we are interested in but not tracing a precise path). Increasing the ocean resolution from GFDL CM2-1deg to CM2.6 shows more modest variance increases than either resolution change for CESM, but is more noticeably positive in the western boundary current regions, tracing the Gulf Stream to an extent. In the bottom panels of Fig. 10, we compute *R* for the full 20 years as well as just for the first 10 years of CM2.6 vs. CM2-1deg output, as a rough test of the robustness of results computed from different periods. The patterns in the two bottom panels resemble each other visually, suggesting that *R* results are relatively robust, at least by the standards of a rough visual test, as long as the averaging period is not too short.

## Discussion

### Increases in atmospheric model resolution

In this section, we compare models having identical ocean components and resolution but with differing atmospheric grid spacing, in an attempt to isolate the effect of decreasing atmospheric model grid spacing on high-frequency precipitation variance. This effect of atmospheric model grid spacing can be assessed between EC-Earth low and EC-Earth high model pairs, both with oceanic grid spacing of 1/12° and with atmospheric grid spacings of 60 and 15.6 km, respectively. The impact of atmospheric model grid spacing can also be investigated using the CESM low and CESM mixed model runs, with 1° ocean models coupled to a 1° and a 0.25° atmosphere respectively. By comparing across the spectra shown in Fig. 9, their mid-frequency band (0.01–0.5 cycles/day) integrated variances (Table 3), and the global differences in variance between different model grid spacings in Fig. 10, we are able to glean some generalized trends in precipitation variance due to atmospheric model grid spacing.

The EC-Earth models are shown in the top panel of Fig. 9 over the Kuroshio region. There is a moderate increase of variance with an increase in resolution from EC-Earth low to EC-Earth high at frequencies higher than 0.2 cycles/day, with a difference between the two models in the mid-frequency band that is not consistent across the entire band. Nevertheless, the integrated sum of precipitation variance over the middle frequency band is about 15% higher in EC-Earth high compared to EC-Earth low.

The spectra of CESM low and CESM mixed are shown in Fig. 9b for the Gulf Stream region. The increase in variance from the low to the mixed model run is significant and generally holds over all frequencies, with the exception of the seasonal cycle and its third harmonic (of 3 cycles/year or 0.00822 cycles/day). The mid-frequency variance sum increases by about 93% from CESM low to CESM mixed. Yet, despite this large increase in variance from CESM low to CESM mixed, CMORPH still has 20% more variance in the mid-frequency band (Table 3). The global distribution of the difference in variance between CESM low and CESM mixed is shown in the top-right panel of Fig. 10. The map shows striking differences in the tropics and equatorial regions. However, it is known that CESM mixed (as well as CESM high) greatly overestimates precipitation at such latitudes, especially over the average Pacific ITCZ position from about 5°–10° N (Small et al. 2014). We thus attribute the large discrepancy in variance between CESM low and CESM mixed in those regions to this overestimation error. The green arrows in the subplots of Fig. 10 point to the subtropical and temperate regions in or downstream from the Gulf Stream and Kuroshio western boundary currents, which have noticeably higher variance increases between these two model runs than other regions at those latitudes.

**Fig. 14:**
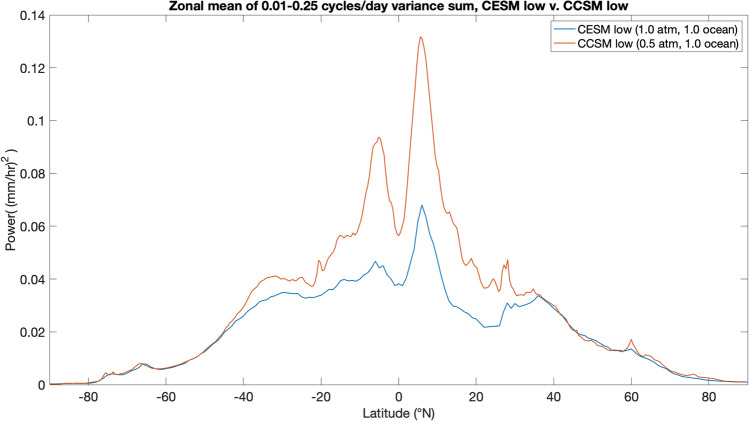
Zonal means of 0.01–0.25 cycles/day variance sums for CESM low and CCSM low

We can also refer to Fig. 9 to compare the CCSM low model (0.5° atmosphere and 1° ocean) and CESM low (1° atmosphere and 1° ocean), although CCSM low's lower-frequency sampling of once every 2 days does not allow comparison at daily periods. In general, it appears that CCSM low has slightly lower variance than CESM low at many frequencies, which opposes the behaviors described above, in which increasing atmospheric resolution also increases variance. This finding is specific to the Gulf Stream and Kuroshio regions, however; CCSM does on average have more variance (integrated between 0.01 and 0.25 cycles/day) for tropical latitudes (Fig. 14).

The general trend we observe in this study is that increasing atmospheric model resolution, which moves closer to resolving mesoscale atmospheric processes, increases precipitation variance, though the specific behavior across models varies and requires further research. It is possible that differences in convection parameterization across grid spacings could be a factor in this trend. The increase in variance in the EC-Earth models is moderate when atmospheric model grid spacing changes (with the control ocean model grid spacing fixed at a value of 1/12°), while there is a much sharper effect with changing the atmospheric model grid spacing in the CESM model runs (with the control ocean model grid spacing being relatively coarse). Both of the free-running EC-Earth models as well as the ERA5 reanalysis tuned to observational data display significantly less variance than CMORPH, although the data assimilation in ERA5 does move the results closer to CMORPH. Particularly relevant is comparison of EC-Earth high with rain gauge data, which has a very high temporal resolution compared to the satellite observation datasets used here. A notable feature in Figs. 5 and 8 is the steeper roll-off in EC-Earth frequency spectra at high frequencies relative to rain gauge results.

**Fig. 15:**
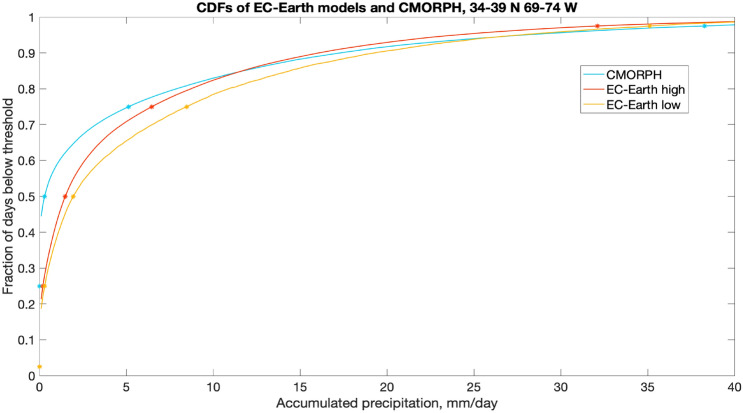
CDF plot focusing on the EC-Earth models in a subregion of the Gulf Stream region

Some previous research (Chen and Dai 2019; Trenberth et al. 2003) has examined the intensity, frequency, and duration of precipitation. A notable finding from these previous studies was that the simulations tend to show too many low-intensity rain events and too few high-intensity rain events—this phenomenon is known as the “drizzle effect”. The drizzle effect is present in all of the models presented in this paper (Figs. 6 and 7), although it may be less prominent with increasing variance; EC-Earth high has less of a drizzle effect than EC-Earth low (Fig. 15), and ERA5 has less than EC-Earth high. However, the existence of a drizzle effect in EC-Earth high is of interest, because the drizzle effect is typically associated with coarse model resolution simulations, in which individual peaks/troughs in low resolution precipitation accumulation time series get flattened into an average in order to yield the same amount of total precipitation. An animation (not shown) was made to further examine this effect; precipitation structures in EC-Earth high are just as complex as would be expected from a model of that resolution, but they covered a noticeably larger area and lacked the precipitation extremes found in TRMM, thus yielding the drizzle effect.

Low-resolution atmospheric models are unable to fully respond to the small-scale variability in high-resolution ocean models (e.g., Hewitt et al 2017; Roberts et al. 2018; Vecchi et al. 2019), and there is some evidence in this study to corroborate this claim. Comparing the precipitation results associated with an atmospheric model resolution increase from CESM low (1°) to CCSM low (0.5°) to CESM mixed (0.25°) in this paper implies that a refinement in resolution to 0.25° yields a "jump step" in high-frequency precipitation greater than is seen in refinements from 1° to 0.5°. Further, the variance in non-tropical regions aligns well with CMORPH for the 0.25° resolution in CESM at both ocean model resolutions, while all 0.5° and coarser atmospheric models significantly underestimate CMORPH-level variance (typically by almost half). Tropical rainfall accumulation (with effects visible indirectly in Fig. 10) is known to be too high compared to observations in CESM high and CESM mixed (Small et al. 2014). It appears that the variance in CESM low to CESM mixed (Fig. 5) is also too high relative to observations. Results from CCSM high are harder to interpret due to the lack of high-frequency model output, but we speculate (from plots not shown) that the 0.5° atmosphere also underestimates variance at most frequencies.

In our results, the two EC-Earth model runs behave similarly in the regions studied. That is, increasing the atmospheric resolution over a high-resolution ocean component has a small effect on precipitation variance. In contrast, comparing the CESM mixed and CESM low runs (which differ in atmospheric resolution, but which both have a low-resolution 1° ocean), we observe a noticeable increase in variance from the lower to the higher atmospheric model resolution.

### Increases in oceanic model resolution

The effect of increases in oceanic model resolution can be examined with both the CESM mixed and CESM high pairing (0.25° atmosphere with 1° and 0.1° ocean, respectively) and the GFDL model suite (0.5° atmosphere with 1°, 0.25°, and 0.1° ocean). The control atmospheric model resolutions differ between the CESM pair and the GFDL model runs as well, such that a comparison between the GFDL and CESM groups provides insight as to whether a high-resolution atmospheric model may be able to better take into account the effects of a high-resolution ocean model.

In the majority of regions we analyzed (Figs. 9, 10, Table 3), there is a small but significant increase in variance from CESM mixed to CESM high across most frequencies. In the Gulf Stream focus region depicted in Fig. 9b, the variance increase with the ocean resolution increase is about 15%. CESM high compares well to CMORPH throughout most frequencies in Fig. 9, but tapers off a bit more than CMORPH does at frequencies higher than 0.2 cycles/day. The spatial comparison of mid-frequency variance between CESM mixed and CESM high (Fig. 10, top left) demonstrates that CESM high has a noticeable Gulf Stream signature (noted by a green arrow in the figure).

Within the GFDL models (Fig. 9c), a small variance increase with increased ocean model resolution is present, but it is weaker than in the CESM case, where the atmospheric model resolution is higher. Over the Northwest Pacific Ocean region, CM2.5 does not have noticeably more mid-frequency variance than in CM2-1deg (as seen in Fig. 9 and Table 3), suggesting that the change from CM2-1deg to CM2.5 provides no significant change in precipitation variance. CM2.6 has about 10% more variance in comparison to CM2.5, indicating that the resolution increase between 0.25° and 0.1° has a greater impact on variance than does the resolution increase from 1° to 0.25°. Still, CM2.6 lies far from observations, with CMORPH having 81% more variance than CM2.6 in this region. The difference between CM2-1deg and CM2.6 is also shown in the map in the bottom left panel of Fig. 10. Some ocean current signatures are visible in downstream regions like the Northwest Pacific, although changes in variance directly over western boundary currents are not as strong as in the CESM ocean resolution change.

Both the CESM and GFDL model families show a moderate increase in variance even if ocean model resolution increase is substantial (from 1° to 0.1°). Based on the maps in Fig. 10 along with results from Sect. 6.1 on atmospheric model resolution increases, it appears that the increase in ocean model resolution has a more pronounced effect on the CESM models, where a variance increase can be clearly seen over strong ocean currents. This observation that the GFDL models show less variance increase due to ocean model resolution increase than do the CESM runs may indicate that high-resolution atmospheric models (having grid spacings of 0.25° or finer) are better able to “feel” ocean model resolution changes, at least with respect to precipitation variance. Some climatic effects are known to occur with an increase in atmospheric model resolution from 1° to 0.5° (Kirtman et al. 2012; Siqueira and Kirtman 2016; Zhang et al. 2021), but the changes that occur in that case do not appear to greatly improve the high-frequency precipitation variance behavior examined here.

### Maps of variance increases

The relative differences, as defined by Eq. (1), between CESM and GFDL simulations having different model resolutions, are given in Fig. 10. Saturation levels of color shading (darkest red or blue) in Fig. 10 indicate that one model has at least 4 times more variance in the mid-frequency band than the other model does. Based on both the top and bottom plots in Fig. 10, changing the ocean model grid spacing from 1.0° to 0.1° in two separate model families (CESM and GFDL) creates a greater variance increase in regions near western boundary currents than in other oceanic regions. In general, there is more variance in the tropics, and localized saturation-level differences there may be related to spatial differences in simulated precipitation formation (the spatial gradient of integrated mid-frequency variance is very large near coastlines). The bottom two plots of Fig. 10, which display the same calculation for 20-year and 10-year subsets of the GFDL CM2.6 vs. CM-1deg comparison, demonstrate that the results do not depend qualitatively on model output duration. Arrows point to the approximate regions of western boundary currents that we have focused on in this paper; note that the maps of variance increases with changes in ocean model resolution show a stronger effect in these areas compared to surrounding regions (for instance, the path of the Gulf Stream is traced on the map comparing CESM high and CESM mixed models).

### Data assimilation

The ERA5 reanalysis uses the same atmospheric component (IFS) as the free-running EC-Earth models, allowing us to assess the impact of data assimilation. ERA5 shows moderately greater variance compared to the free-running EC-Earth high at most frequencies (Fig. 9a). The increase is most evident at frequencies above 0.2 cycles/day, and is less evident at lower frequencies. Mid-frequency variance in ERA5 is about 27% higher in the Kuroshio region than EC-Earth high (Table 3), while CMORPH still has roughly 25% more total variance in these bands than ERA5.

The Navy ESPC model also uses data assimilation, and its variance at most frequencies is close to that of CMORPH, especially in regions near the Gulf Stream (Fig. 8). It contains somewhat less variance than CMORPH, but its relative similarity to ERA5 provides additional evidence that models incorporating data assimilation can output a more accurate level of high-frequency precipitation variance compared to free-running models.

### Speculative results amongst GEOS high-resolution models

Within the GEOS models, a change in both atmospheric and ocean resolutions (doubling each) does increase variance significantly, and the effect is somewhat more pronounced at high frequencies above 1 cycle/day (Fig. 9a). The relatively high amount of variance observed in these models (Figs. 8, 9) suggests the importance of model spatial resolution in precipitation variance spectra. These models have a high output frequency (1 h), and generally show greater variance than CMORPH, TRMM, and the rain gauges. The greater variance in the GEOS models could be due to the time series covering only one annual cycle, and the increase in variance after both atmospheric and oceanic model resolutions are increased is consistent with the trends we see in other model groupings. It is important to note that the GEOS high model was run for less than 1 year, and we therefore remain cautious when interpreting the results.

## Summary and conclusions

We have examined high-frequency precipitation variance in several coupled atmosphere–ocean modeling systems. Twelve of the systems are free-running (non-data-assimilative). We also examine the US Navy Earth System Prediction Capability model, a weather forecast system employing data assimilation in both fluids. We compare the modeled results to estimates of precipitation from rain gauges, the TRMM and CMORPH satellite-derived products, and the ECMWF ERA5 atmospheric model reanalysis. We employ three analysis tools–frequency spectra, cumulative distribution functions, and an investigation of irregular sub-daily fluctuations. Covey et al. (2018) employed the latter analysis in an examination of satellite products and low-resolution climate models.

Here we apply the Covey et al. (2018) analysis to rain gauges and higher-resolution models. Whereas Covey et al. (2018) found that zonally averaged irregular sub-daily fluctuations in lower-resolution climate models were insufficiently energized relative to satellite-derived observational products, we find here that the higher-resolution US Navy ESPC weather forecast model exhibits zonally averaged irregular sub-daily fluctuations lying closer to those in satellite products. The relatively close match of the Navy EPSC forecast model to the satellite product results demonstrates the value added by data assimilation. The GEOS/ECCO simulations exhibit zonally averaged irregular sub-daily fluctuations that are higher than those in the satellite products. The fact that irregular sub-daily fluctuations contain most of the precipitation variance (Covey et al. 2018) argues for saving precipitation more frequently—at, for instance, hourly intervals—than is normally done in climate model simulations.

The Covey et al. (2018) analysis cannot be applied to most of the modeling products used in this paper, because their precipitation values are not output at subdaily intervals. We therefore focus on frequency spectra and cumulative distribution functions (CDFs) of the precipitation outputs in the latter sections of this paper. The focus of our frequency spectra analyses is on time scales of 2–100 days, i.e., time scales that are relatively high-frequency in the context of climate modeling. Overall, our work suggests that refining the grid spacing of either atmospheric or oceanic model components will increase high-frequency precipitation variance, and bring it closer to values seen in observational products. For the models examined here, the grid spacing of the atmospheric model has the larger impact. Ocean model grid spacing does have a measurable impact, especially when the atmospheric model grid spacing is relatively fine (less than 0.5°). The CDFs reveal that the “drizzle effect”—i.e., the tendency for models to rain more frequently than is seen in observations—is seen in all of the model systems examined here.

## Data Availability

https://doi.org/10.7302/fn7r-hq31.

## Appendix

First, we display zonal mean irregular sub-daily fluctuations from individual years of CMORPH observations alongside the zonal mean computed from 1998 to 2014 (Fig. 11). The values of zonal means in individual years are generally less than those over the 17-year period. However, the differences between results from individual years and the entire 17 year record are not as large as the differences between the means of irregular sub-daily fluctuations and other components shown in Figs. 2 and 3, or between means of irregular sub-daily fluctuations computed from CMORPH and some of the other models. This implies that the large differences between the irregular sub-daily fluctuations computed from the 1 year output of the GEOS simulations and the 17-year output of CMORPH seen earlier are not primarily due to the short duration of the GEOS simulation records.

Ratios of variance in irregular sub-daily fluctuations to total variance, in rain gauge records and outputs of CMORPH, Navy ESPC, GEOS low, and GEOS high at the rain gauge locations, are displayed in Fig. 12. Consistent with the zonal average results shown in Fig. 11, the ratios computed from individual years of CMORPH at rain gauge locations are lower than the ratios computed from the 17-year CMORPH record. Similarly, the ratios computed from individual years of the NOAA gauge data are lower than the ratios computed from the 4-year NOAA records. The ratio computed from the 1-min SPURS2 record is higher than the ratio computed from the hourly SPURS2 record. The ratio computed from individual years of CMORPH is usually lower than the ratios computed from individual years of the NOAA rain gauges, Navy ESPC, and the GEOS models. The GEOS results lie closer to the rain gauge results than the results computed from other 1-year outputs of CMORPH.

Next, we define the regions shown in Fig. 4. The first region is over the ocean surrounding the Gulf Stream; the combined area of all points within the bounding boxes of 30°–33°N, 77°–80°W; 30°–34°N, 73°–77°W; 34°–36°N, 74°–75.5°W; 34°–39°N, 69°–74°W; 36°–42°N, 60°–69°W; and 37°–43°N, 50°–60°W. The second region is directly over the Kuroshio current: the combined area of bounding boxes 25.5°–27°N, 122°–127.5°E; 27°–28.5°N, 125°–129°E; 28.5°–31°N, 126.5°–132°E; 29°–33°N, 132°–140°E; 32°–34.5°N, 137°–143°E; and 33°–37°N, 141°–153°E. A greater Northwest Pacific region was also examined: the rectangular area defined by 25°–50°N, 120°E-170°W. This latter region was chosen specifically in reference to analysis of the GFDL model runs, as a statistically significant increase in variance was present over this larger region but not directly over the main part of the Kuroshio. For comparisons against rain gauges, we use two regions where data is available and which are close enough to the ocean to ascertain direct oceanic influences on precipitation patterns. The Atlantic coast region is relatively close to the Gulf Stream; model and estimate datasets there used a region of 25°–38°N, 74°–82°W, and rain gauges (from NOAA Local Climatological Data) were located in Miami FL, Melbourne FL, Jacksonville FL, Savannah GA, Charleston SC, Wilmington NC, and Norfolk VA. The rain gauge used in the SPURS-II experiment was located in the Pacific Ocean near 10°N, 125°W; models we compare against it were averaged over the region 7°–12°N, 122°–127°W.

Next we examine the effect on precipitation variance from spatial resolution only. A variance spectrum made with original model output from the GEOS low model, and a variance spectrum derived from the GEOS low output spatially averaged as 16-point mean accumulation for each hour in the same region, are shown in Fig. 13. The results demonstrate that smoothing out the spatial features of precipitation lowers the spectral variance, consistent with the expectation that higher-resolution models have more variance.

The regional analyses in Figs. 5, 8, and 9 may suggest that the increased atmospheric model resolution in CCSM low vs. CESM low does not yield increased high-frequency precipitation variance. An analysis that includes more tropical regions demonstrates the expected increase with increased resolution. The zonal mean of variance integrated over the 0.01–0.25 cycles/day band for the CESM low and CCSM low models contains more variance over tropical latitudes in CCSM, consistent with CCSM low's higher atmospheric model resolution (Fig. 14). The analysis in Fig. 14 employs the 0.01–0.25 cycles/day frequency band, rather than the 0.01–0.5 cycles/day band used in most of the other analyses, because CCSM low output was only sampled every two days.

There are some regions where the CDF behavior of the EC-Earth models better matches CMORPH in the high-resolution model. Comparison of CDFs for the EC-Earth models along with CMORPH in a portion of the Gulf Stream (34°-39°N, 69°-74°W) indicates that the higher-resolution model run has both increased variance and a CDF balanced more towards major precipitation events over drizzle (Fig. 15). This behavior of EC-Earth in the Gulf Stream region contrasts with the behavior at the SPURS-II region (Fig. 5), at which the higher-resolution model run does not display either increased variance nor a CDF balanced more towards major precipitation events.

## Code availability

https://doi.org/10.7302/fn7r-hq31.
